# An aggregation-induced emission platform for efficient Golgi apparatus and endoplasmic reticulum specific imaging†

**DOI:** 10.1039/d1sc03932f

**Published:** 2021-10-05

**Authors:** Peihong Xiao, Ke Ma, Miaomiao Kang, Luyi Huang, Qian Wu, Nan Song, Jinyin Ge, Dan Li, Jianxia Dong, Lei Wang, Dong Wang, Ben Zhong Tang

**Affiliations:** Center for AIE Research, Shenzhen Key Laboratory of Polymer Science and Technology, Guangdong Research Center for Interfacial Engineering of Functional Materials, College of Materials Science and Engineering, Shenzhen University Shenzhen 518060 China wangd@szu.edu.cn; Key Laboratory of Optoelectronic Devices and Systems of Ministry of Education and Guangdong Province, College of Physics and Optoelectronic Engineering, Shenzhen University Shenzhen 518060 China; Department of Chemistry, Hong Kong Branch of Chinese National Engineering Research, Center for Tissue Restoration and Reconstruction, The Hong Kong University of Science and Technology Clear Water Bay Kowloon Hong Kong 999077 China tangbenz@ust.hk; Shenzhen Institute of Molecular Aggregate Science and Engineering, School of Science and Engineering, The Chinese University of Hong Kong Shenzhen, 2001 Longxiang Boulevard, Longgang District Shenzhen City Guangdong 518172 China tangbenz@cuhk.edu.cn; Key Laboratory of Molecular Biology for Infectious Diseases (Ministry of Education), Institute for Viral Hepatitis, Department of Infectious Diseases, The Second Affiliated Hospital, Chongqing Medical University Chongqing 400010 China; Department of Clinical Pharmacy, West China Hospital of Sichuan University Chengdu 610041 Sichuan Province China

## Abstract

As two important subcellular organelles in eukaryotic cells, the Golgi apparatus (GA) and endoplasmic reticulum (ER) have recently captivated much interest due to their considerable importance in many biofunctions and role as critical biomarkers for various diseases. The development of efficient GA- and ER-specific probes is of great significance, but remains an appealing yet significantly challenging task. Herein, we reported for the first time the construction of an aggregation-induced emission (AIE) platform for GA and ER fluorescent probes, termed as AIE-GA and AIE-ER, by facile synthesis and simple functionalization. Their excellent targeting specificity to GA or ER, remarkable photostability, high brightness, and low working concentration make AIE-GA and AIE-ER significantly impressive and superior to commercially available probes. Moreover, molecular docking calculations are performed to validate the targeting mechanism of the two AIE probes.

## Abstract 2

As two important subcellular organelles in eukaryotic cells, the Golgi apparatus (GA) and endoplasmic reticulum (ER) have recently captivated much interest due to their considerable importance in many biofunctions and role as critical biomarkers for various diseases.

## Graphical abstract

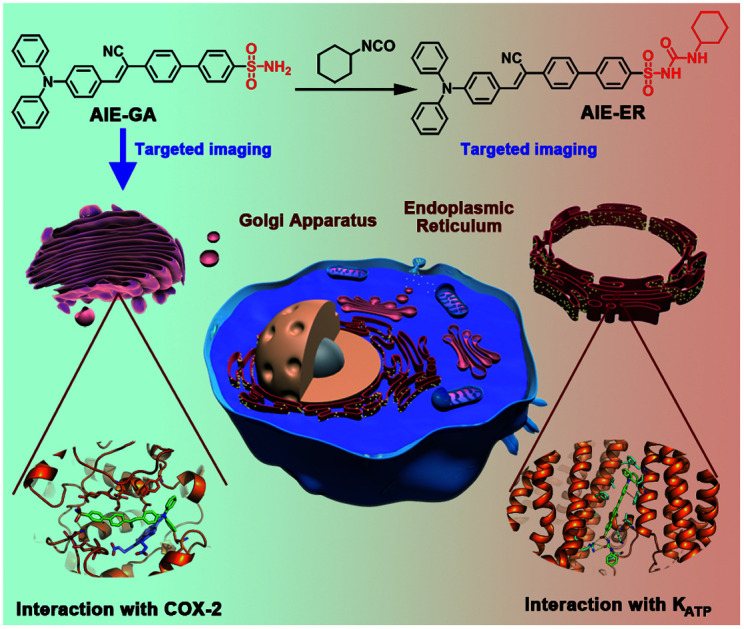

## Introduction

The Golgi apparatus (GA) and endoplasmic reticulum (ER) are two considerably important and closely related subcellular organelles in eukaryotic cells.^1^ As the largest cellular organelle, the ER is responsible for protein synthesis and folding, lipid metabolism, calcium storage and redox homeostasis.^2–4^ The GA is well known as a “post-office” and mainly participates in processing and modifying various proteins and lipids synthesized by the ER, and then correctly classifying and transporting them to intracellular and extracellular destinations.^5–7^ It has been demonstrated that the morphological changes and dysfunction of the GA and ER can lead to many diseases, such as cancer,^8,9^ vascular disease,^10^ type II diabetes,^11^ Alzheimer's disease^12^ and Huntington's disease.^13^ Due to the important signal pathways to trigger cancer death induced by GA and ER stress, both the GA and ER have recently emerged as promising drug targets for cancer treatment.^14–17^ Therefore, *in situ* GA- and ER-specific targeting and imaging are greatly significant for real-time observation of the intracellular dynamic process and in-depth understanding of the disease pathogenesis. The rapid development of the fluorescence imaging technique has provided an opportunity for *in situ* dynamic observation and monitoring of subcellular organelles, by virtue of its various intrinsic advantages including superb sensitivity, rapidity, real-time and on-site responsiveness and noninvasiveness.^18–20^ Although some types of GA (such as Golgi Tracker Red/Green derived from boron-dipyrromethene) and ER (such as DiOC5(3), DiOC6(3), and ER-Tracker Red/Green) probes have been commercialized, the current situation is still far from ideal. Those previously reported probes have their respective and collective drawbacks including modest selectivity, tedious synthetic routes and complicated purification process.^21–23^ In particular, they often suffer from photobleaching at working concentrations, along with the aggregation-caused quenching (ACQ) phenomenon at slightly higher concentrations during the staining process.

Given the circumstances, as a completely opposite effect of ACQ, the emergence of aggregation-induced emission (AIE) has triggered the development of state-of-the-art fluorescence imaging.^24^ AIE luminogens (AIEgens) are non-emissive or weakly emissive due to intramolecular motions when dissolved in solvents, but present a strongly boosted emission upon aggregation or binding to biomarkers resulting from such restricted intramolecular motions. This characteristic could enable AIEgens to serve as ideal “turn-on” fluorescent probes and show significant advantages in the field of biological analysis and imaging.^25^ Recently, AIEgens have made great progress and achievement in staining the cell membrane,^26^ mitochondria,^27^ lysosomes,^28^ lipid droplets,^29^ and nucleus.^30^ However, there are limited examples of AIE probes that are capable of staining the GA^31^ and ER.^32^ They were designed based on different AIE-active fluorophores with tedious synthesis and complicated purification. What's worse, the specificity of some probes is far from ideal with only a moderate Pearson's correlation coefficient. Organelle-targeted fluorescent probes based on an AIE platform are more efficient and cost-effective, and may offer an opportunity to observe organelles interplay in live samples. Despite these intriguing advantages, to the best of our knowledge, such an AIE platform allowing both GA and ER targeted staining has yet to be reported, and remains a very challenging task.

**Fig. 1:**
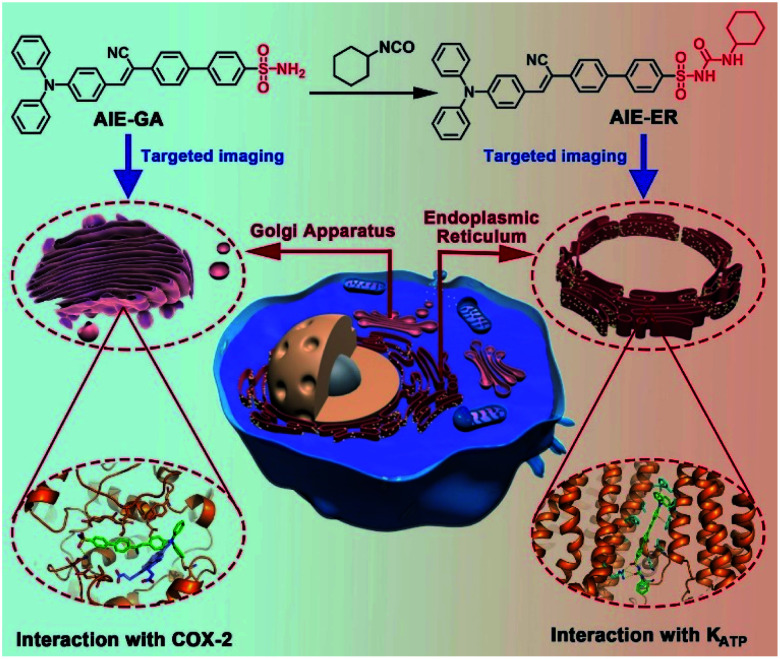
Schematic illustration of specific Golgi apparatus (GA) and endoplasmic reticulum (ER) targeting by AIEgens with one fluorophore through one-step conversion.

In this contribution, we report for the first time the rational design and facile synthesis of GA and ER probes using one AIE fluorophore through simple late-stage functionalization. On the basis of the ACQ-to-AIE transformation which is achieved by subtle structural regulation, phenylsulfonamide and glibenclamide skeletons are linked to the fluorophores, yielding the GA- and ER-specific targeting probes, respectively (Fig. 1). Cellular imaging outputs reveal that these AIE-featured probes exhibit superior performance to the corresponding ACQ-active ones in terms of biocompatibility, specificity, and photostability. Moreover, the targeting mechanisms of the two AIE probes were elucidated by molecular docking assay.

## Results and discussion

### Design and synthesis

**Fig. 2:**
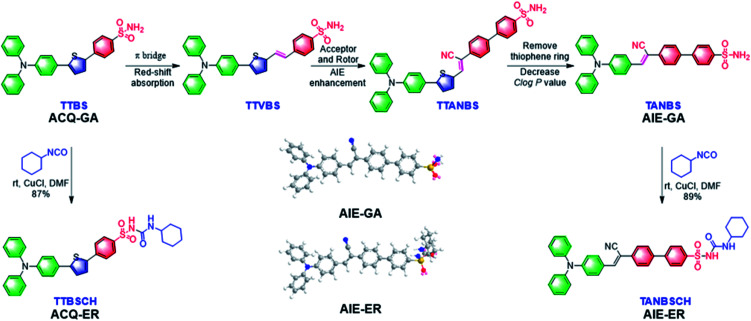
The rational design of GA and ER probes.

In the molecular design, the propeller-shaped triphenylamine (TPA) fragment is used as both an electron donor (D) and rotor. The phenylsulfonamide unit is utilized as the Golgi targeting moiety due to its strong binding ability with cyclooxygenase-2 (COX-2), which is an inducing enzyme that expresses and accumulates in the Golgi apparatus.^33,34^ Additionally, the glibenclamide moiety that can bind to the ATP-sensitive potassium channel (K_ATP_) on the ER membrane^35,36^ is employed to achieve ER-targeting. TTBS, TTVBS, TTANBS and TANBS are designed as GA probes (Fig. 2). Aiming to elongate the π-conjugation length and increase the D–A strength, double bond and cyano units are introduced to form TTVBS and TTANBS, respectively. The orderly increased D–A strength ranging from TTBS and TTVBS to TTANBS indicates the increasing bathochromic shift of both absorption and emission in this order. With the increasing number of benzene rings, the hydrophobicity of TTBS, TTVBS and TTANBS is raised, which can be proved from the enlarged *C* log *P* values (7.81, 8.35, and 8.87, respectively). Considering that the high log *P* value is unfavourable for achieving GA-targeting,^34^ taking into account of GA targeting ability and AIE properties, the thiophene ring was removed from TTANBS to give TANBS with a decreased *C* log *P* value of 7.10, which could be a potential AIE probe for staining GA. Once these probes achieved GA targeting ability, it was anticipated that ER targeting probes can be easily constructed by simple functionalization of the GA probes. To verify these hypotheses, we started with the synthesis and characterization of these GA probes. As shown in Scheme S1,† TTBS was facilely prepared by two-step Suzuki reactions. The first step was the synthesis of compound **1** by Suzuki reaction of 4-bromo-*N*,*N*-diphenylaniline with 2,5-dibromothiophene in the presence of a Pd catalyst. The second Suzuki coupling reaction proceeded between compound **1** and a 4-acid pinacol ester to afford TTBS. TTVBS was simply synthesized by Horner–Wadsworth–Emmons (HWE) reaction between compound **2** and diethyl (4-sulfamoylbenzyl) phosphonate mediated by NaH. TTANBS was also obtained by a two-step reaction: a Knoevenagel condensation between compound **2** and 2-(4-bromophenyl) acetonitrile, followed by a Suzuki coupling with 4-sulfamoylphenylboronic acid pinacol ester. TANBS was simply prepared by the same procedure as TTANBS, starting from corresponding aldehyde compound **5**. With TTBS and TANBS in hand, two ER probes TTBSCH and TANBSCH were prepared by a one-step conversion using a Cu catalyst, respectively. All the GA probes and ER probes are characterized by NMR spectra and mass spectra (Fig. S1–S21†).

### Photophysical properties

**Fig. 3:**
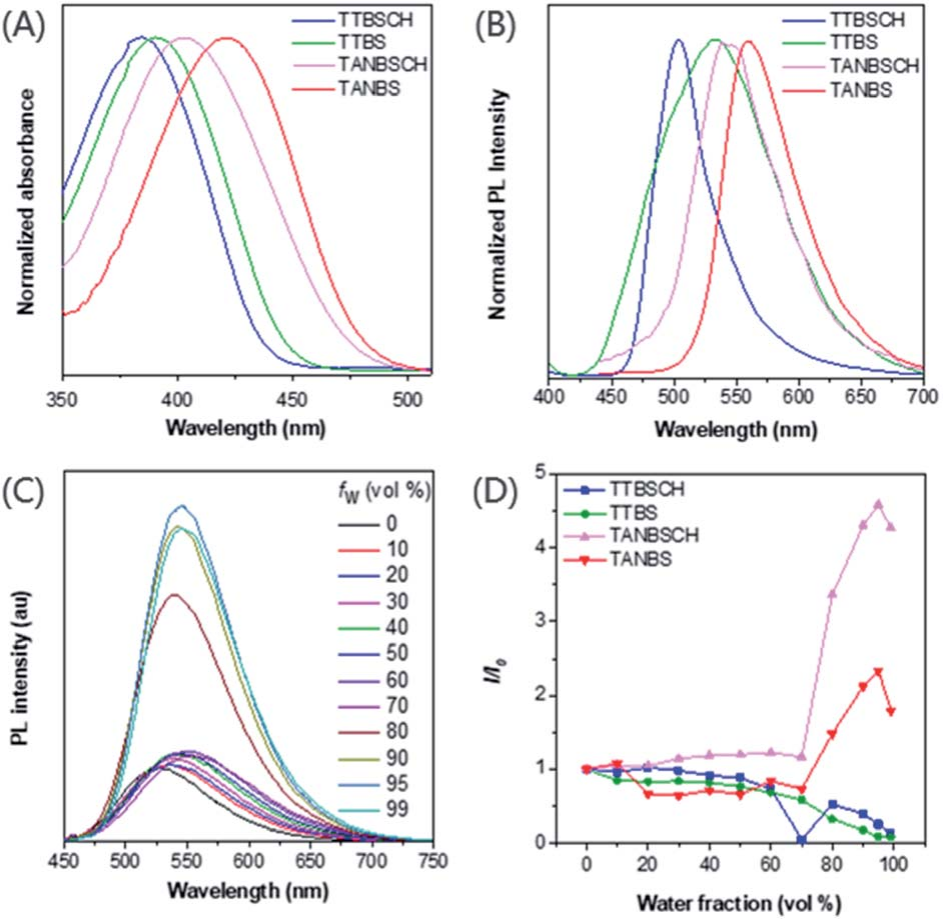
(A) Normalized absorption spectra of TTBSCH, TTBS, TANBSCH and TANBS in the DMSO solution, concentration = 10 × 10^−6^ M. (B) Normalized PL spectra of TTBSCH, TTBS, TANBSCH and TANBS in the solid state. (C) PL spectra of TANBSCH (10 × 10^−6^ M) in THF/water mixtures with different water fractions (*f*_w_). (D) The plot of the relative emission intensity (*I*/*I*_0_) *versus* the composition of the THF/water mixture of TTBSCH, TTBS, TANBSCH and TANBS, where *I*_0_ was the PL intensity at *f*_w_ = 0.

The photophysical properties of GA probes (TTBS, TTVBS, TANBS and TTANBS) and ER probes (TTBSCH and TANBSCH) were measured by absorption and fluorescence emission spectra. The maximum absorption peaks of GA probes are located at 393, 405, 420 and 442 nm in DMSO solution (Fig. 3A and S24†). Meanwhile, a similar bathochromic shift was also found for fluorescence emission in both DMSO solution and solid state (Fig. 3B and S24, and Table S1†). Meanwhile, for ER probes, the maximum absorption peaks blue-shifted to 384 and 403 nm compared with the corresponding GA probes. To investigate the AIE properties of GA probes, fluorescence intensities of TTBS in different solvent mixtures were recorded (Fig. 3D, S22 and S23†). It is unanticipated that the fluorescence intensities of TTBS decreased dramatically in THF/water, DMSO/water and MeCN/water mixtures with the increase of water fraction, strongly indicating the ACQ feature of TTBS. The introduction of a double bond in TTVBS elongated the conjugation and made the absorption and emission slightly red-shift, and it led to a slight improvement of the AIE property (Fig. S25†). In sharp contrast, the fluorescence intensities of TTANBS were enhanced to 1.69 fold at a water fraction of 90%, clearly demonstrating that TTANBS is a typical AIE-active probe. When the thiophene ring was removed, the maximum absorption and emission peaks of TANBS blue-shifted to 420 and 560 nm due to the weakened D–A strength. With the increase of water fraction, the fluorescence intensities of TANBS were boosted and reached maxima at a water fraction of 95%, due to the formation of aggregation. The ER probe TANBSCH showed better performance in terms of AIE properties compared to the GA probe TANBS, which may be ascribed to the more twisted conformation and stronger hydrogen bond interactions in the aggregate/solid state, while another ER probe, TTBSCH, transformed from the corresponding TTBS was still an ACQ molecule (Fig. 3D).

### Cell imaging studies

**Fig. 4:**
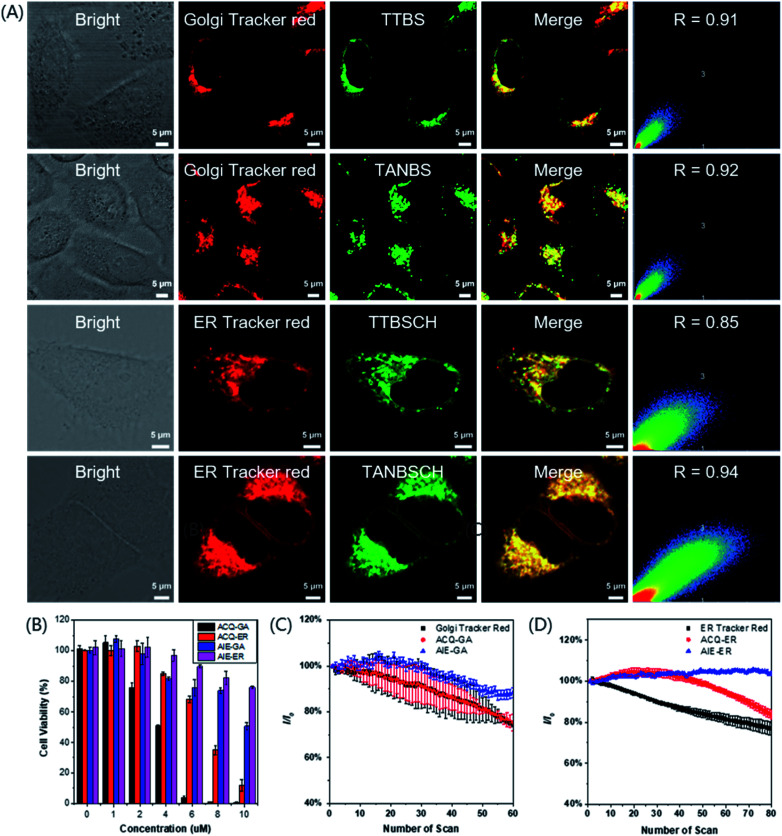
(A) Confocal microscopy imaging of HeLa cells labeled with TTBS (2 × 10^−6^ M) and the colocalization with Golgi-Tracker Red (333 μg ml^−1^); Pearson's coefficient (*R*) = 0.91; TANBS (2 × 10^−6^ M) and its colocalization with Golgi-Tracker Red (333 μg ml^−1^); Pearson's coefficient (*R*) = 0.92; TTBSCH (1 × 10^−6^ M) and its colocalization with ER-Tracker Red (1 × 10^−6^ M); Pearson's coefficient (*R*) = 0.85; TANBSCH (1 × 10^−6^ M) and its colocalization with ER-Tracker Red (1 × 10^−6^ M); Pearson's coefficient (*R*) = 0.94. Scale bar = 5 μM. (B) Cell viability of HeLa cells stained with different concentrations of ACQ-GA, ACQ-ER, AIE-GA and AIE-ER. (C) Loss in fluorescence of HeLa cells stained with ACQ-GA, AIE-GA and Golgi Tracker Red with increasing the number of scans of laser irradiation. Concentration: 2 × 10^−6^ M (ACQ-GA), 2 × 10^−6^ M (AIE-GA) and 333 μg ml^−1^ (Golgi Tracker Red); ACQ-GA (green channel: *λ*_ex_ = 405 nm, *λ*_em_ = 415–600 nm); AIE-GA (green channel: *λ*_ex_ = 405 nm, *λ*_em_ = 500–700 nm); Golgi Tracker Red (red channel: *λ*_ex_ = 589 nm, *λ*_em_ = 600–700 nm). (D) Loss in fluorescence of HeLa cells stained with ACQ-ER, AIE-ER and ER Tracker Red with increasing the number of scans of laser irradiation. Concentration: 1 × 10^−6^ M (ACQ-ER), 1 × 10^−6^ M (AIE-ER) and 1 × 10^−6^ M (ER Tracker Red); ACQ-ER (green channel: *λ*_ex_ = 405 nm, *λ*_em_ = 450–650 nm); AIE-ER (green channel: *λ*_ex_ = 405 nm, *λ*_em_ = 450–650 nm); ER Tracker Red (red channel: *λ*_ex_ = 543 nm, *λ*_em_ = 570–650 nm).

To evaluate the targeting ability of the GA probes, a commercially available red emission dye, Golgi Tracker Red, was used for colocalization staining combined with different GA probes (Fig. 4A and S26†). TTBS (0.91) and TANBS (0.92) had a higher Pearson's colocalization coefficient than TTVBS (0.78) and TTANBS (0.68). This result is in good accordance with the expected molecular structural variation trend. With the increasing *C* log *P* values of TTBS (7.81), TTVBS (8.35) and TTANBS (8.87), their Pearson's colocalization coefficient with Golgi Tracker Red decreased in turn, which indicated that high log *P* values are indeed detrimental to Golgi targeting. Thanks to the low *C* log *P* value of TANBS (7.10), the Pearson's colocalization value between TANBS and Golgi Tracker Red was up to 0.92. Control experiments were also investigated to confirm whether these GA probes could stain other subcellular organelles, especially the mitochondrion. Mito-Tracker Red was used to stain the mitochondrion of HeLa cells co-cultured with TTBS and TANBS, which displayed rather low Pearson's colocalization coefficients (0.51 and 0.44, respectively, Fig. S27†).

In order to verify our hypothesis that different subcellular organelles can be targeted by simple functionalization of a targeting fluorescent probe, TTBSCH and TANBSCH were applied for colocalization experiments with ER Tracker Red (a commercially available red emission ER dye) to verify the ER identification ability. The AIE-active probe TANBSCH had a higher Pearson's colocalization coefficient (0.94) than the ACQ-active probe TTBSCH (0.85), indicating the superior ER-targeting specificity of TANBSCH (Fig. 4A). In the staining process, it was observed that the brightness of HeLa cells stained with ER Tracker Red was inconsistent. Moreover, commercially available ER Tracker Red tended to form aggregates during the staining process, and thus there were always several spots with particular bright red emission in the cytoplasm.^32*b*^ In a sharp contrast, the brightness of AIE-active TANBSCH was uniform and there was only a little bright spot in the cytoplasm, which indicated that TANBSCH had a better ER identification ability than ER Tracker Red. To make these probes more concise and easier to distinguish, we finally named the GA probes TTBS and TANBS as ACQ-GA and AIE-GA, respectively. The two ER probes TTBSCH and TANBSCH were termed as ACQ-ER and AIE-ER, respectively. Furthermore, the targeting ability of AIE-GA and AIE-ER was successfully verified by respectively co-staining with Golgi Tracker Red and ER Tracker Red in both 4T1 cells and A549 cells (Fig. S28†). Huvec cells were selected as normal cells to evaluate the targeting ability of AIE-GA and AIE-ER. As shown in Fig. S29,† AIE-GA can also image the Golgi apparatus in Huvec cells, but the Pearson's colocalization coefficient decreased to 0.68, which is much lower than that in HeLa cells (0.92). This is mainly due to the fact that COX-2 is more expressed in cancer cells than in normal cells. While AIE-ER can target the Endoplasmic reticulum of Huvec cells with Pearson's colocalization coefficients up to 0.86.

### Cytotoxicity and photostability

The low cytotoxicity of fluorescent probes is necessary for long-term bioimaging and tracking. To evaluate the cytotoxicity, MTT (3-(4,5-dimethyl-2-thiazolyl)-2,5-diphenyltetrazolium bromide) assay was employed to detect the cell viability of HeLa cells after the 24 h incubation of ACQ-GA, AIE-GA, ACQ-ER and AIE-ER with different concentrations. The cell viability had an obvious decline with the increasing amount of AIE-active or ACQ-active probes (Fig. 4B). But the cytotoxicity of AIE-GA and AIE-ER probes was much lower than that of the corresponding ACQ-GA and ACQ-ER probes. Moreover, the cells incubated with AIE-active probes maintained good cell viabilities (>75%) even at a high probe concentration of 8 μM, which was 4-fold or 8-fold higher than the proper staining concentration (2 μM for GA; 1 μM for ER). Therefore, compared with the ACQ-active probes, the AIE-active probes had much lower cytotoxicity.

Photostability is another important factor for long-term bioimaging and tracking due to its influence on the imaging quality.^37,38^ The emission intensity of AIE-GA only decreased by about 10% after 60 irradiation scans. In sharp contrast, the fluorescence signal loss of ACQ-GA and Golgi Tracker Red was up to 25% upon light irradiation under the same conditions, demonstrating the superior photostability of AIE-GA to those of ACQ-GA and Golgi Tracker Red (Fig. 4C). A similar photostability was observed between ACQ-ER and ER Tracker Red with about 20% fluorescence signal loss, while AIE-ER was very stable under light irradiation (Fig. 4D).

### Docking calculations and verification of the response mechanism

**Fig. 5:**
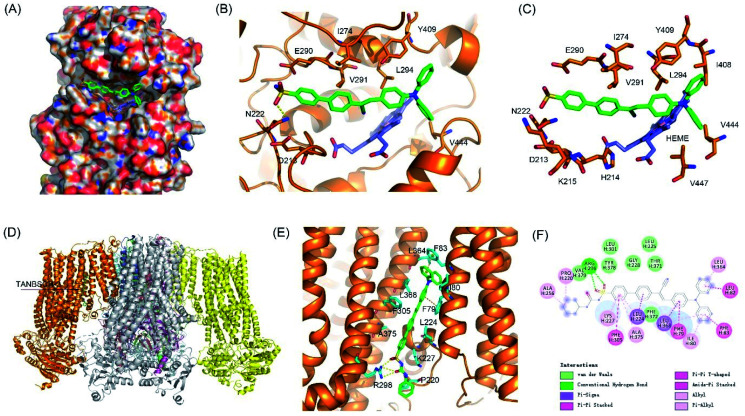
Putative binding modes of the AIE-GA – COX-2 and AIE-ER – KATP channel complexes. (A) Surface view of the AIE-GA – COX-2 complex. (B and C) Interactions between AIE-GA and COX-2. AIE-GA and HEME were represented as green and blue sticks, respectively. (D) Structural model of KATP channel complexes with AIE-ER. (E) Detailed interactions between AIE-ER and SUR1. (F) Two-dimensional interaction map of SUR1 complexes with AIE-ER.

To validate the targeting mechanism of AIE-GA and AIE-ER, molecular docking calculations were performed using the Autodock Vina program. Previous reports have shown that compounds with the phenylsulfonamide group selectively bound to the Golgi protein cyclooxygenase-2 (COX-2).^39,40^ Thus, the crystal structure of COX-2 was chosen as the putative target of the AIE-GA probe. AIE-GA bound to the cyclooxygenase catalytic site of COX-2. The phenylsulphonamide ring was buried in a hydrophobic groove surrounded by H214, K215, N222, I274, E290, and V291. The triphenylamine moiety bound to a cavity adjacent to HEME and formed a T shaped π–π interaction with Y409 (Fig. 5A–C). These results demonstrated that the AIE-GA probe had a favourable binding ability to interact with COX-2.

Meanwhile, ER targeting is achieved by binding to the subunit SUR1 domain of K_ATP_ channels, which are widely distributed on the ER membrane.^41^ As we expected, AIE-ER could specifically bound to the SUR1 in a similar mode to ER Tracker Red and glibenclamide. AIE-ER fully occupied the well-tailored pocket formed by the residues F79, I80, F83, L224, K227, R298, F305, L364, L368, and A375. Specifically, AIE-ER interacted with the SUR1 domain through hydrogen bonds with R298 and π–π stacking interactions with F79, F83, and F305 (Fig. 5D–F). Taken together, the AIE-ER probe can bind to the SUR1 domain with high binding affinity through several strong interactions.

## Conclusions

In summary, we develop for the first time two novel fluorescent probes, namely AIE-GA and AIE-ER, on the basis of one fluorophore with AIE characteristics for GA and ER localization. Both of these two AIE probes have a much better biocompatibility, photostability, and Pearson's colocalization coefficient than the corresponding ACQ-GA and ACQ-ER probes, respectively. Additionally, compared with the traditional commercially available Golgi Tracker Red and ER Tracker Red, these two AIE probes exhibit the intrinsic advantages of simple synthesis, easy purification, high sensitivity and excellent photostability, making them beneficial to *in situ* long-time tracking and imaging. This study not only provides new insights into the design of different organelle specific probes with AIE-activity, but also offers two probes with great transformation potential for dynamic tracking and monitoring of the GA and ER, which may provide an avenue for the study of diseases related to these two subcellular organelles.

## Experimental section

### Materials and instruments

All solvents and chemicals, unless specifically stated, were of analytical grade, purchased commercially and used without further purification. ^1^H and ^13^C spectra were measured on Bruker ARX 500 (or 600) NMR spectrometers using DMSO-*d*_6_ and CDCl_3_ as the deuterated solvent, respectively. Tetramethylsilane (TMS; *δ* = 0 ppm) was used as the internal standard. High-resolution mass spectra (HRMS) were recorded on a Finnegan MAT TSQ 7000 Mass Spectrometer System. UV-vis absorption spectra were taken on a PerkinElmer Lambda 950 spectrophotometer. PL spectra were recorded on an Edinburgh FS5 fluorescence spectrophotometer and Milton Roy Spectronic 3000 array spectrophotometer. The absolute PLQY was determined using a Hamamatsu quantum yield spectrometer, C11347 Quantaurus QY. The cell viability was examined by MTT assay. The cellular fluorescence images were taken using a confocal laser scanning microscope (CLSM, ZEISS-LSM880). Compounds **1**,^42^**2**,^43^**3**,^44^ and **6** (ref. 45) were synthesized according to a literature method.

### Synthesis of TTBS

Compound **1** (60 mg, 0.15 mmol), 4-sulfamoylphenylboronic acid pinacol ester (50 mg, 0.18 mmol), potassium carbonate (82 mg, 0.59 mmol) and Pd (PPh_3_)_4_ (17 mg, 0.02 mmol) were added into 4 ml THF and 1 ml H_2_O in a round bottom flask with a condenser under nitrogen, and the reaction mixture was heated to reflux at 80 °C overnight with stirring. After cooling to room temperature, the mixture was extracted with DCM (10 ml *×* 3). The organic phase was combined and washed with water, then dried over anhydrous Na_2_SO_4_. After concentrated under reduced pressure, the crude product was purified by silica-gel column chromatography (gradient eluent: DCM/MeOH = 100 : 1 → 20 : 1) to afford TTBS (66 mg, 92% yield) as a yellow powder. ^1^H NMR (600 MHz, DMSO-*d*_6_, *δ*): 7.86 (d, *J* = 8.9 Hz, 2H), 7.84 (d, *J* = 9.0 Hz, 2H), 7.67 (d, *J* = 3.9 Hz, 1H), 7.63–7.59 (m, 2H), 7.47 (d, *J* = 3.8 Hz, 1H), 7.40 (s, 2H), 7.36–7.30 (m, 4H), 7.11–7.05 (m, 6H), 7.01–6.97 (m, 2H); ^13^C NMR (151 MHz, DMSO-*d*_6_, *δ*): 147.19, 146.77, 144.19, 142.52, 139.75, 136.66, 129.69, 127.07, 126.78, 126.58, 126.51, 125.18, 124.44, 124.24, 123.59, 122.81. HRMS (ESI-TOF, *m*/*z*) calcd for C_28_H_23_N_2_O_2_S_2_^+^ [M + H]^+^: 483.11955, found 483.12014.

### Synthesis of TTVBS

To a solution of compound **2** (36 mg, 0.10 mmol) and compound **3** (31 mg, 0.10 mmol) in 2 ml DMF was added NaH (4.8 mg, 0.20 mmol, dissolved in 2 ml DMF) dropwise at 0 °C under a nitrogen atmosphere. The resulting solution was allowed to stir overnight at room temperature. Then the solvent was removed under reduced pressure, the crude product was purified by silica-gel column chromatography (gradient eluent: petroleum ether/EtOAc = 100 : 1 → 10 : 1) to afford TTVBS (48 mg, 94% yield) as a yellow powder. ^1^H NMR (600 MHz, DMSO-*d*_6_, *δ*): 7.79 (d, *J* = 8.2 Hz, 2H), 7.74 (d, *J* = 8.3 Hz, 2H), 7.61–7.56 (m, 3H), 7.38 (d, *J* = 3.8 Hz, 1H), 7.36–7.31 (m, 6H), 7.26 (d, *J* = 3.8 Hz, 1H), 7.10–7.08 (m, 2H), 7.07–7.05 (m, 4H), 7.00–6.97 (m, 3H); ^13^C NMR (151 MHz, DMSO-*d*_6_, *δ*): 147.10, 146.78, 143.07, 142.46, 140.34, 140.14, 129.70, 129.70, 129.56, 127.26, 126.46, 126.45, 126.14, 125.79, 124.62, 124.43, 123.59, 122.86. HRMS (ESI-TOF, *m*/*z*) calcd for C_30_H_25_N_2_O_2_S_2_^+^ [M + H]^+^: 509.1352, found 509.1352.

### Synthesis of **4**

Compound **2** (300 mg, 0.84 mmol) was dissolved in 9 ml ethanol, 4-bromophenylacetonitrile (168 mg, 0.84 mmol) and NaOEt (5.7 mg, 0.08 mmol) were added, and then the mixture was stirred at room temperature for 2 hours. After the reaction completed, the crude product was purified by recrystallization from EtOH to give compound **4** (344 mg, 76% yield) as an orange solid. ^1^H NMR (600 MHz, chloroform-*d*, *δ*): 7.59–7.49 (m, 8H), 7.31–7.29 (m, 4H), 7.24 (d, *J* = 4.0 Hz, 1H), 7.14 (d, *J* = 7.9 Hz, 4H), 7.10–7.05 (m, 4H); ^13^C NMR (151 MHz, chloroform-*d*, *δ*): 149.80, 148.50, 147.09, 135.83, 134.74, 134.54, 133.15, 132.18, 129.42, 126.98, 126.64, 124.95, 123.63, 122.76, 122.73, 122.63, 122.55, 118.06, 105.37. HRMS (ESI-TOF, *m*/*z*) calcd for C_31_H_22_BrN_2_S^+^ [M + H]^+^: 533.0682, found 533.0687.

### Synthesis of TTANBS

Compound **4** (150 mg, 0.28 mmol), 4-sulfamoylphenylboronic acid pinacol ester (95 mg, 0.34 mmol), potassium carbonate (155 mg, 1.12 mmol) and Pd (PPh_3_)_4_ (33 mg, 0.03 mmol) were added into 8 ml THF and 2 ml H_2_O in a round bottom flask with a condenser under nitrogen, and the reaction mixture was heated to reflux at 80 °C overnight with stirring. After cooling to room temperature, the mixture was extracted with DCM (10 ml × 3). The organic phase was combined and washed with water, then dried over anhydrous Na_2_SO_4_. After being concentrated under reduced pressure, the crude product was purified by silica-gel column chromatography (gradient eluent: DCM/MeOH = 100 : 1 → 20 : 1) to afford TTANBS (140 mg, 82% yield) as an orange red powder. ^1^H NMR (500 MHz, DMSO-*d*_6_, *δ*): 8.37 (s, 1H), 7.96–7.91 (m, 4H), 7.91–7.84 (m, 4H), 7.77 (d, *J* = 4.1 Hz, 1H), 7.67 (d, *J* = 8.7 Hz, 2H), 7.58 (d, *J* = 4.0 Hz, 1H), 7.42 (m, 2H), 7.38–7.34 (m, 4H), 7.32–7.28 (m, 1H), 7.13 (d, *J* = 8.7 Hz, 1H), 7.10–7.09 (m, 4H), 7.00 (d, *J* = 8.6 Hz, 2H); ^13^C NMR (151 MHz, DMSO-*d*_6_, *δ*): 148.54, 147.96, 146.56, 143.26, 142.15, 138.64, 136.94, 135.80, 135.65, 133.50, 129.74, 127.73, 127.09, 127.03, 126.36, 126.16, 125.95, 124.75, 123.89, 123.47, 122.28, 118.13, 104.34. HRMS (ESI-TOF, *m*/*z*) calcd for C_37_H_27_N_3_O_2_S_2_ [M]: 609.1545, found 609.1542.

### Synthesis of TANBS

Compound **6** (100 mg, 0.22 mmol), 4-sulfamoylphenylboronic acid pinacol ester (75 mg, 0.27 mmol), potassium carbonate (122 mg, 0.88 mmol) and Pd (PPh_3_)_4_ (25 mg, 0.02 mmol) were added into 4 ml THF and 1 ml H_2_O in a round bottom flask with a condenser under nitrogen, and the reaction mixture was heated to reflux at 80 °C overnight with stirring. After cooling to room temperature, the mixture was extracted with DCM (10 ml × 3). The organic phase was combined and washed with water, then dried over anhydrous Na_2_SO_4_. After being concentrated under reduced pressure, the crude product was purified by silica-gel column chromatography (gradient eluent: petroleum ether/EtOAc = 20 : 1 → 2 : 1) to afford TANBS (85 mg, 73% yield) as a yellow powder. ^1^H NMR (600 MHz, DMSO-*d*_6_, *δ*): 8.00 (s, 1H), 7.93 (d, *J* = 3.5 Hz, 4H), 7.91–7.88 (m, 2H), 7.88–7.84 (m, 4H), 7.43 (s, 2H), 7.41–7.37 (m, 4H), 7.20–7.17 (m, 2H), 7.17–7.13 (m, 4H), 6.97 (d, *J* = 8.8 Hz, 2H); ^13^C NMR (151 MHz, DMSO-*d*_6_, *δ*): 149.61, 146.01, 143.25, 142.33, 142.22, 138.60, 134.25, 130.93, 129.91, 127.67, 127.10, 126.36, 126.11, 126.04, 125.64, 124.75, 119.95, 118.45, 105.60, 39.94, 39.92, 39.80, 39.66, 39.52, 39.38, 39.24, 39.10. HRMS (ESI-TOF, *m*/*z*) calcd for C_33_H_25_N_3_O_2_S [M − H]^+^: 526.1667, found 526.1592.

### Synthesis of TANBSCH

To a stirred solution of TANBS (53 mg, 0.10 mmol) and CuCl (1.0 mg, 0.01 mmol) in DMF (2.0 ml) under a nitrogen atmosphere, cyclohexyl isocyanate (19 mg, 0.15 mmol) was added dropwise. The reaction was stirred for 24 hours at room temperature. Then the solvent was removed under reduced pressure, and the crude product was purified by silica-gel column chromatography (gradient eluent: DCM/MeOH = 100 : 1 → 20 : 1) to afford TANBSCH (58 mg, 89% yield) as a yellow powder. ^1^H NMR (500 MHz, DMSO-*d*_6_, *δ*): 10.43 (s, 1H), 8.01 (s, 1H), 8.00–7.94 (m, 4H), 7.91–7.84 (m, 6H), 7.40 (t, *J* = 7.8 Hz, 4H), 7.18 (t, *J* = 7.5 Hz, 2H), 7.15 (d, *J* = 7.8 Hz, 4H), 6.96 (d, *J* = 8.8 Hz, 2H), 6.39 (d, *J* = 7.5 Hz, 1H), 1.68–1.64 (m, 2H), 1.61–1.57 (m, 2H), 1.50–1.46 (m, 1H), 1.28–1.16 (m, 3H), 1.15–1.08 (m, 3H); ^13^C NMR (126 MHz, DMSO-*d*_6_, *δ*): 150.56, 149.63, 146.00, 143.42, 142.44, 138.35, 134.47, 130.94, 129.89, 129.82, 127.96, 127.79, 127.09, 126.09, 126.06, 125.64, 124.74, 119.93, 118.42, 105.54, 48.13, 32.28, 24.97, 24.20; HRMS (ESI-TOF, *m*/*z*) calcd for C_40_H_36_N_4_O_3_S [M − H]^+^: 651.2424, found 651.2437.

### Synthesis of TTBSCH

To a stirred solution of TTBS (49 mg, 0.10 mmol) and CuCl (1.0 mg, 0.01 mmol) in DMF (2.0 ml) under a nitrogen atmosphere, cyclohexyl isocyanate (19 mg, 0.15 mmol) was added dropwise. The reaction was stirred for 24 hours at room temperature. Then the solvent was removed under reduced pressure and the crude product was purified by silica-gel column chromatography (gradient eluent: DCM/MeOH = 100 : 1 → 20 : 1) to afford TTBSCH (53 mg, 87% yield) as a yellow powder. ^1^H NMR (600 MHz, DMSO-*d*_6_, *δ*): 10.38 (s, 1H), 7.92–7.85 (m, 4H), 7.71 (d, *J* = 3.9 Hz, 1H), 7.63 (d, *J* = 8.7 Hz, 2H), 7.49 (d, *J* = 3.9 Hz, 1H), 7.36–7.33 (m, 4H), 7.12–7.08 (m, 2H), 7.09–7.06 (m, 4H), 6.99 (d, *J* = 8.7 Hz, 2H), 6.36 (d, *J* = 7.9 Hz, 1H), 1.69–1.64 (m, 2H), 1.60–1.57 (m, 2H), 1.49–1.46 (m, 1H), 1.27–1.16 (m, 3H), 1.15–1.08 (m, 3H); ^13^C NMR (151 MHz, DMSO-*d*_6_, *δ*): 147.28, 146.76, 144.67, 139.43, 138.29, 137.81, 129.71, 128.24, 127.30, 126.97, 126.56, 125.09, 124.47, 124.33, 123.62, 122.77, 48.13, 32.30, 24.99, 24.23. HRMS (ESI-TOF, *m*/*z*) calcd for C_35_H_34_N_3_O_3_S_2_ [M + H]^+^: 608.2036, found 608.2042.

### Cell lines

The human epithelioid cervical carcinoma cells (HeLa), adenocarcinomic human alveolar basal epithelial cells (A549), and mouse breast cancer cells (4T1) were purchased from the Institute of Cell Biology (Shanghai, China). Cells were all propagated in T-75 flasks cultured at 37 °C under a humidified 5% CO_2_ atmosphere in DMEM medium (GIBCO/Invitrogen, Camarillo, CA, USA), which were supplemented with 10% fetal bovine serum (FBS, Biological Industry, Kibbutz Beit Haemek, Israel) and 1% penicillin–streptomycin (10 000 U mL^−1^ penicillin and 10 mg ml^−1^ streptomycin, Solarbio Life Science, Beijing, China).

### Cell imaging and confocal colocalization

HeLa cells, A549 cells and 4T1 cells were cultured in Dulbecco's Modified Eagle Medium (DMEM) containing 10% fetal bovine serum (FBS) and antibiotics (100 units mL^−1^ penicillin and 100 mg ml^−1^ streptomycin) in a 5% CO_2_ humidity incubator at 37 °C. For Golgi apparatus staining, cells were first incubated with ACQ-GA and AIE-GA, respectively. Then Golgi Tracker Red was added and incubated at 37 °C for 30 min. For endoplasmic reticulum staining, cells were first incubated with ER Tracker Red, then ACQ-ER and AIE-ER were added and incubated, respectively, at 37 °C for 30 min, and the medium was removed and the cells were rinsed with phosphate buffered saline (PBS) three times and then imaged under a confocal microscope (CLSM, ZEISS-LSM880). The excitation was 405 nm for ACQ-GA, AIE-GA, ACQ-ER and AIE-ER, 543 nm for ER Tracker Red, and 633 nm for Golgi Tracker Red.

### Cytotoxicity study

Cells were seeded in 96-well plates (Costar, IL, U.S.A.) at a density of 5000 cells per well. After overnight culturing, the medium in each well was replaced by 100 μL of fresh medium containing different concentrations of ACQ-GA, AIE-GA, ACQ-ER and AIE-ER. After 24 h incubation, 10 μL of MTT solution (5 mg ml^−1^ in PBS) was added into each well and incubated for 4 h. After a further 30 min of incubation under light irradiation, in which another array of plates in the dark was used as the control, 100 μL of DMSO was added to each well and then vibrated for 15 min. The absorption of each well at 595 nm was recorded using a plate reader (PerkinElmer Victor3TM). Each trial was performed with 6 parallel wells. The relative cell viability (%) was calculated using the following formula: cell viability (%) = mean absorbance value of the treatment group-blank/mean absorbance value of the control blank × 100%.

### Photostability

The cells were imaged using a confocal microscope (CLSM, ZEISS-LSM880) and analysed using ZEN 2009 software (Carl Zeiss). The concentrations were 2 × 10^−6^ M for ACQ-GA and AIE-GA probes, and 1 × 10^−6^ M for ACQ-ER and AIE-ER probes. ACQ-GA, AIE-GA, ACQ-ER and AIE-ER were excited at 405 nm, Golgi-Tracker Red was excited at 633 nm, and ER-Tracker Red was excited at 543 nm (2% laser power). The resulting curve represents the bleaching rate.

### Molecular docking assay

The receptors were extracted from the crystal structure of COX-2 (PDB ID: 5IKR) and K_ATP_ channel (PDB ID: 6JB1), respectively. The macromolecule and small molecules (AIE-ER and AIE-GA) were prepared using Auto Dock Tools. Each inhibitor was docked into the corresponding putative binding pocket by Auto Dock Vina. Visualization of the calculation results was performed using pymol.

## Data availability

All experimental supporting data and procedures are available in the ESI.†

## Author contributions

Peihong Xiao: conceptualization, investigation, writing – original draft, writing – review & editing. Ke Ma: investigation, writing – review & editing. Miaomiao Kang: investigation. Luyi Huang: formal analysis. Qian Wu: formal analysis, investigation. Nan Song: writing – review & editing. Jinyin Ge: investigation. Dan Li: writing – review & editing. Jianxia Dong: writing – review & editing. Lei Wang: writing – review & editing. Dong Wang: supervision, methodology, writing – review & editing. Ben Zhong Tang: supervision, methodology, writing – review & editing.

## Conflicts of interest

There are no conflicts of interest to declare.

## Supplementary Material

SC-012-D1SC03932F-s001

## References

[cit1] Pothukuchi P., Agliarulo I., Russo D., Rizzo R., Russo F., Parashuraman S. (2019). FEBS Lett..

[cit2] Hetz C. (2012). Nat. Rev. Mol. Cell Biol..

[cit3] Clapham D. E. (2007). Cell.

[cit4] Shi Y., Wang S., Wu J., Jin X., You J. (2021). J. Controlled Release.

[cit5] Lodish H., Bark A., Zipersky S. L., Matsudaira P., Baltimore D. and Darnell J., Molecular Cell Biology, W. H. Freeman, New York, 4th ed, 2000

[cit6] Zappa F., Failli M., De Matteis M. A. (2018). Curr. Opin. Cell Biol..

[cit7] Glick B. S., Nakano A. (2009). Annu. Rev. Cell Dev. Biol..

[cit8] Lin J. H., Walter P., Yen T. S. B. (2008). Annu. Rev. Pathol.: Mech. Dis..

[cit9] Preisinger C., Short B., De Corte V., Bruyneel E., Haas A., Kopajtich R., Gettemans J., Barr F. A. (2004). J. Cell Biol..

[cit10] Lu L., Zhou Q., Chen Z., Chen L. (2018). J. Cell. Physiol..

[cit11] Özcan U., Cao Q., Yilmaz E., Lee A.-H., Iwakoshi N. N., Özdelen E., Tuncman G., Görgün C., Glimcher L. H., Hotamisligil G. S. (2004). Science.

[cit12] Eto K., Asada T., Arima K., Makifuchi T., Kimura H. (2002). Biochem. Biophys. Res. Commun..

[cit13] Sbodio J. I., Snyder S. H., Paul B. D. (2018). Proc. Natl. Acad. Sci. U. S. A..

[cit14] Nam J. S., Kang M.-G., Kang J., Park S.-Y., Lee S. J. C., Kim H.-T., Seo J. K., Kwon O.-H., Lim M. H., Rhee H.-W., Kwon T.-H. (2016). J. Am. Chem. Soc..

[cit15] Feng Z., Wang H., Wang S., Zhang Q., Zhang X., Rodal A. A., Xu B. (2018). J. Am. Chem. Soc..

[cit16] Gao P., Pan W., Li N., Tang B. (2019). ACS Appl. Mater. Interfaces.

[cit17] Li H., Zhang P., Luo J., Hu D., Huang Y., Zhang Z.-R., Fu Y., Gong T. (2019). ACS Nano.

[cit18] Johnson I. and Spence M. T. Z., Molecular Probes Handbook, A Guide to Fluorescent Probes and Labeling Technologies, Molecular Probes, Eugene, OR, USA, 2010

[cit19] Zhu H., Fan J., Du J., Peng X. (2016). Acc. Chem. Res..

[cit20] Klymchenko A. S. (2017). Acc. Chem. Res..

[cit21] Pagano R. E., Martin O. C., Kang H. C., Haugland R. P. (1991). J. Cell Biol..

[cit22] Colston J., Horobin R., Rashid-Doubell F., Pediani J., Johal K. (2003). Biotech. Histochem..

[cit23] Cole L., Davies D., Hyde G. J., Ashford A. E. (2000). J. Microsc..

[cit24] Luo J., Xie Z., Lam J. W. Y., Cheng L., Chen H., Qiu C., Kwok H. S., Zhan X., Liu Y., Zhu D., Tang B. Z. (2001). Chem. Commun..

[cit25] Mei J., Leung N. L. C., Kwok R. T. K., Lam J. W. Y., Tang B. Z. (2015). Chem. Rev..

[cit26] Shi H., Liu J., Geng J., Tang B. Z., Liu B. (2012). J. Am. Chem. Soc..

[cit27] Leung C. W. T., Hong Y., Chen S., Zhao E., Lam J. W. Y., Tang B. Z. (2013). J. Am. Chem. Soc..

[cit28] Hu F., Cai X., Manghnani P. N., Kenry, Wu W., Liu B. (2018). Chem. Sci..

[cit29] Wang D., Su H., Kwok R. T. K., Shan G., Leung A. C. S., Lee M. M. S., Sung H. H. Y., Williams I. D., Lam J. W. Y., Tang B. Z. (2017). Adv. Funct. Mater..

[cit30] Yu C. Y. Y., Zhang W., Kwok R. T. K., Leung C. W. T., Lam J. W. Y., Tang B. Z. (2016). J. Mater. Chem. B.

[cit31] Xing X., Jia Y., Zhang J., Wu Z., Qin M., Li P., Feng X., Sun Y., Zhao G. (2021). Sens. Actuators, B.

[cit32] Alam P., He W., Leung N. L. C., Ma C., Kwok R. T. K., Lam J. W. Y., Sung H. H. Y., Williams I. D., Wong K. S., Tang B. Z. (2020). Adv. Funct. Mater..

[cit33] Kurumbail R. G., Stevens A. M., Gierse J. K., McDonald J. J., Stegeman R. A., Pak J. Y., Gildehaus D., iyashiro J. M., Penning T. D., Seibert K., Isakson P. C., Stallings W. C. (1996). Nature.

[cit34] Zhang H., Fan J., Wang J., Zhang S., Dou B., Peng X. (2013). J. Am. Chem. Soc..

[cit35] Phaniraj S., Gao Z., Rane D., Peterson B. R. (2016). Dyes Pigm..

[cit36] Zünkler B. J., Wos-Maganga M., Panten U. (2004). Biochem. Pharmacol..

[cit37] Zhang J., Wang Q., Guo Z., Zhang S., Yan C., Tian H., Zhu W.-H. (2019). Adv. Funct. Mater..

[cit38] Niu G., Zhang R., Gu Y., Wang J., Ma C., Kwok R. T. K., Lam J. W. Y., Sung H. H. Y., Williams I. D., Wong K. S., Yu X., Tang B. Z. (2019). Biomaterials.

[cit39] Wang H., He Z., Yang Y., Zhang J., Zhang W., Zhang W., Li P., Tang B. (2019). Chem. Sci..

[cit40] Wang H., Yang Y., Huang F., He Z., Li P., Zhang W., Zhang W., Tang B. (2020). Anal. Chem..

[cit41] Ding D., Wang M., Wu J.-X., Kang Y., Chen L. (2019). Cell Rep.

[cit42] Jia J., Hu C., Cui Y., Li Y., Wang W., Han L., Li Y., Gao J. (2018). Dyes Pigm..

[cit43] Niu G., Zheng X., Zhao Z., Zhang H., Wang J., He X., Chen Y., Shi X., Ma C., Kwok R. T. K., Lam J. W. Y., Sung H. H. Y., Williams I. D., Wong K. S., Wang P., Tang B. Z. (2019). J. Am. Chem. Soc..

[cit44] Yang L.-M., Lin S.-J., Hsu F.-L., Yang T.-H. (2002). Bioorg. Med. Chem. Lett..

[cit45] Ji C., Yin L., Xie B., Wang X., Li X., Zhang J.-J., Ni J., Li Y. (2016). Synth. Met..

