# Supplementary material for: An aggregation-induced emission platform for efficient Golgi apparatus and endoplasmic reticulum specific imaging
Source: Chem Sci. 2021 Oct 5;12(41):13949–57. doi: 10.1039/d1sc03932f (PMC8549777; doi:10.1039/d1sc03932f)
Supplement: SC-012-D1SC03932F-s001 [file SC-012-D1SC03932F-s001.pdf]

Electronic Supplementary Material (ESI) for Chemical Science.
This journal is © The Royal Society of Chemistry 2021

Supporting Information

An Aggregation-Induced Emission Platform for Efficient Golgi Apparatus and Endoplasmic Reticulum Specific Imaging

Peihong Xiao,‡[ab] Ke Ma,‡[ab] Miaomiao Kang,[ab] Luyi Huang,[e] Qian Wu,[c] Nan Song,[ab] Jinyin Ge,[ab] Dan Li,[ab] Jianxia Dong,[f] Lei Wang,[a] Dong Wang*[a] and Ben Zhong Tang*[cd]

[a] Center for AIE Research, Shenzhen Key Laboratory of Polymer Science and Technology, Guangdong Research Center for Interfacial Engineering of Functional Materials, College of Materials Science and Engineering, Shenzhen University, Shenzhen 518060, China. E-mail: wangd@szu.edu.cn

[b] Key Laboratory of Optoelectronic Devices and Systems of Ministry of Education and Guangdong Province, College of Physics and Optoelectronic Engineering, Shenzhen University, Shenzhen 518060, China

[c] Department of Chemistry, Hong Kong Branch of Chinese National Engineering Research, Center for Tissue Restoration and Reconstruction, The Hong Kong University of Science and Technology, Clear Water Bay, Kowloon, Hong Kong 999077, China. E-mail: tangbenz@ust.hk

[d] Shenzhen Institute of Molecular Aggregate Science and Engineering, School of Science and Engineering, The Chinese University of Hong Kong, Shenzhen, 2001 Longxiang Boulevard, Longgang District, Shenzhen City, Guangdong 518172, China. E-mail: tangbenz@cuhk.edu.cn

[e] Key Laboratory of Molecular Biology for Infectious Diseases (Ministry of Education), Institute for Viral Hepatitis, Department of Infectious Diseases, The Second Affiliated Hospital, Chongqing Medical University, Chongqing, 400010, China

[f] Department of Clinical Pharmacy, West China Hospital of Sichuan University, Chengdu 610041, Sichuan Province, China

‡These authors contributed equally to this work.

Table of Contents

1. **Synthetic route of Golgi apparatus and Endoplasmic Reticulum probes……. 2**
2. **NMR and HRMS spectrum………………………………………………. 3-12**
3. **PL spectra of TTBS in THF/water and DMSO/water mixtures……………... 13**
4. **PL spectra of TTBS in MeCN/water and MeOH/Glycerol mixtures……….. 14**
5. **Normalized absorption and emission spectra of TTVBS and TTANBS…….. 14**
6. **PL spectra of TTVBS and TTANBS in THF/water mixtures………………… 15**
7. **Co-localization experiment of TTVBS, TTANBS and Golgi Tracker Red……15**

8. **Co-localization experiment of TTBS, TANBS and Mito Tracker Red……… 16**

9. **Co-localization experiment of AIE-GA, AIE-ER, Golgi Tracker Red and ER Tracker Red in 4T1 and A549 cells……………………………………….. 16**

10. **Co-localization experiment of AIE-GA, AIE-ER, Golgi Tracker Red and ER Tracker Red in Huvec cells…………………………………………………. 17**

11. **Molecular orbital amplitude plots of the HOMO and LUMO energy levels of ACQ-GA, AIE-GA, ACQ-ER and AIE-ER………………………………… 17**

12. **Optical properties of AIE-GA, AIE-ER, ACQ-GA and ACQ-ER………….. 18**

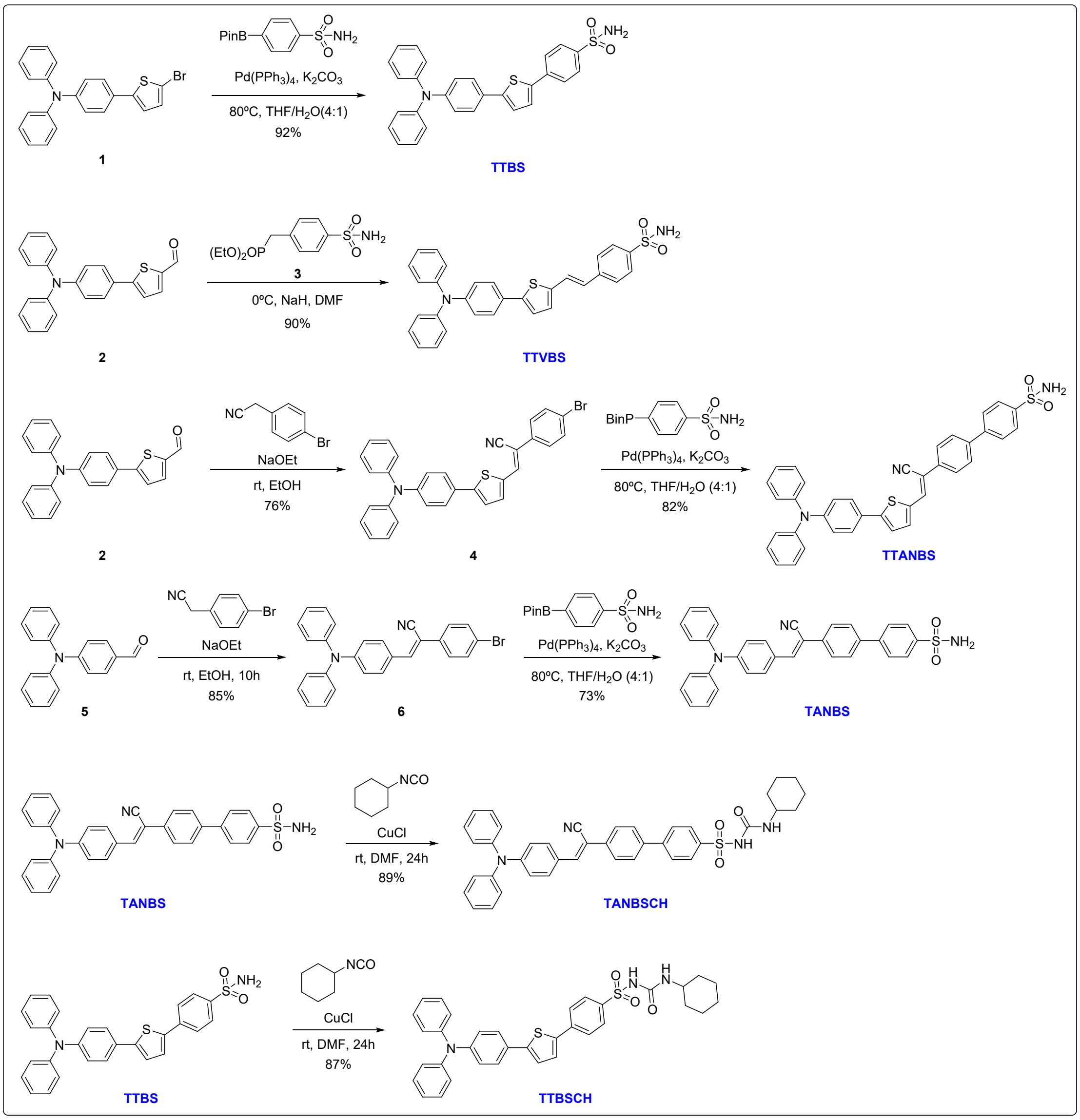

Scheme S1. Synthetic route of Golgi apparatus and Endoplasmic Reticulum probes.

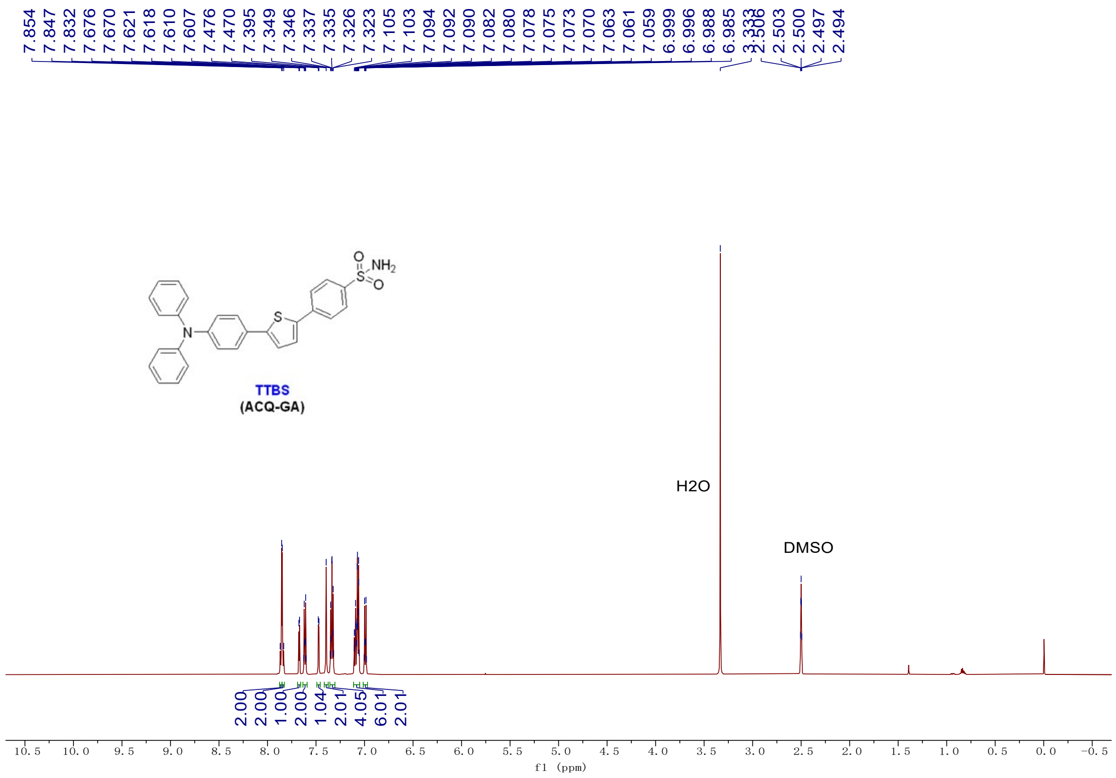

Fig. S1 ^{1}H NMR spectrum of TTBS.

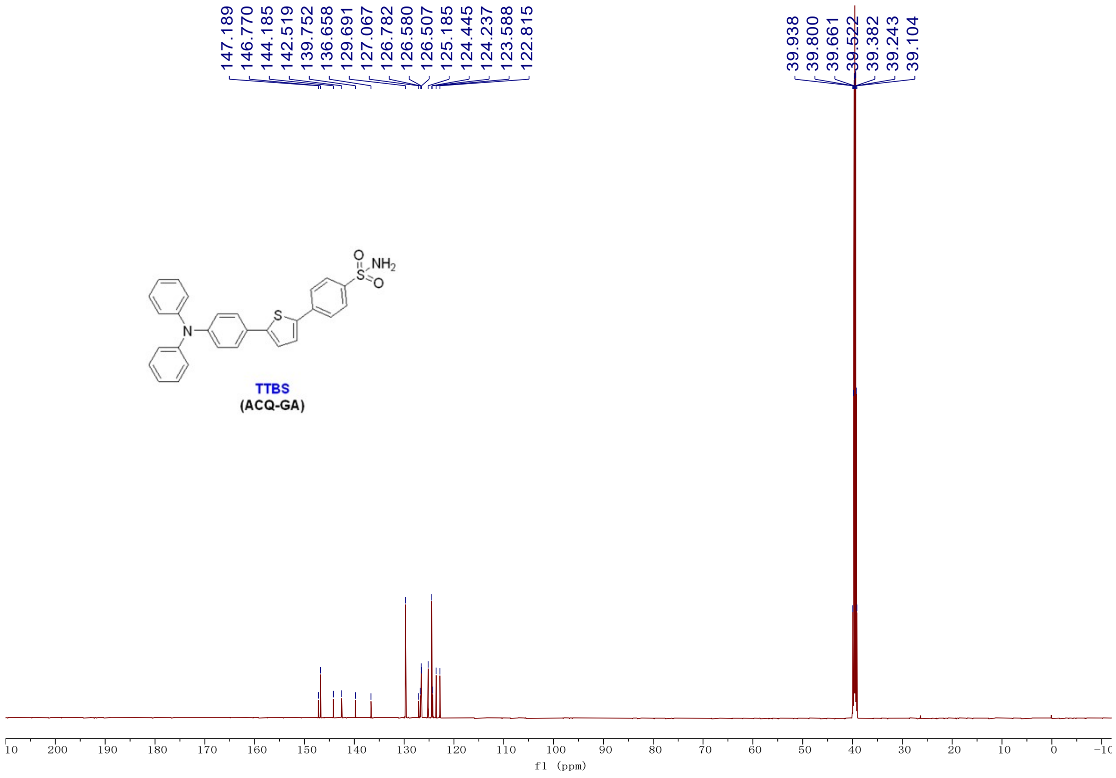

Fig. S2 ^{13}C NMR spectrum of TTBS.

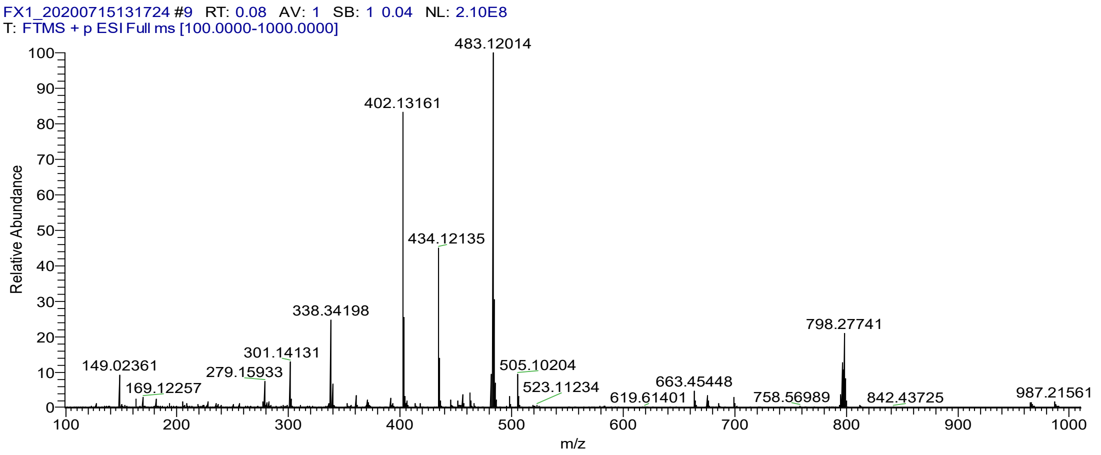

Fig. S3 HRMS spectrum of TTBS.

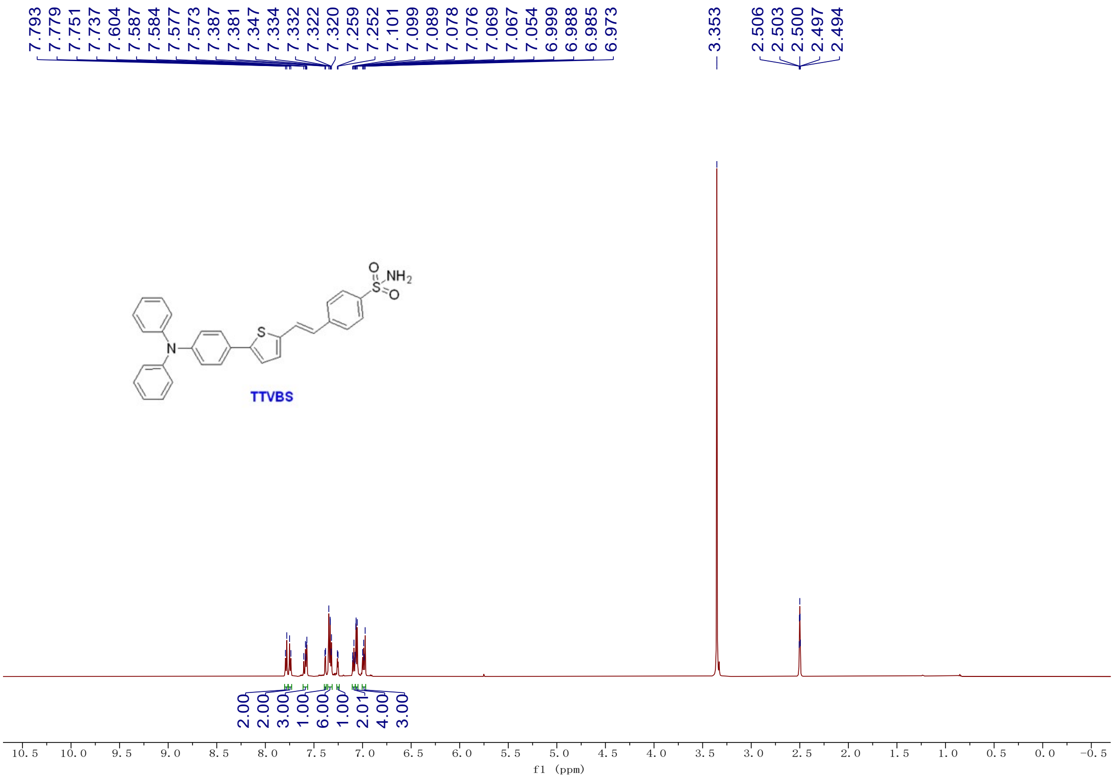

Fig. S4 ^{1}H NMR spectrum of TTVBS.

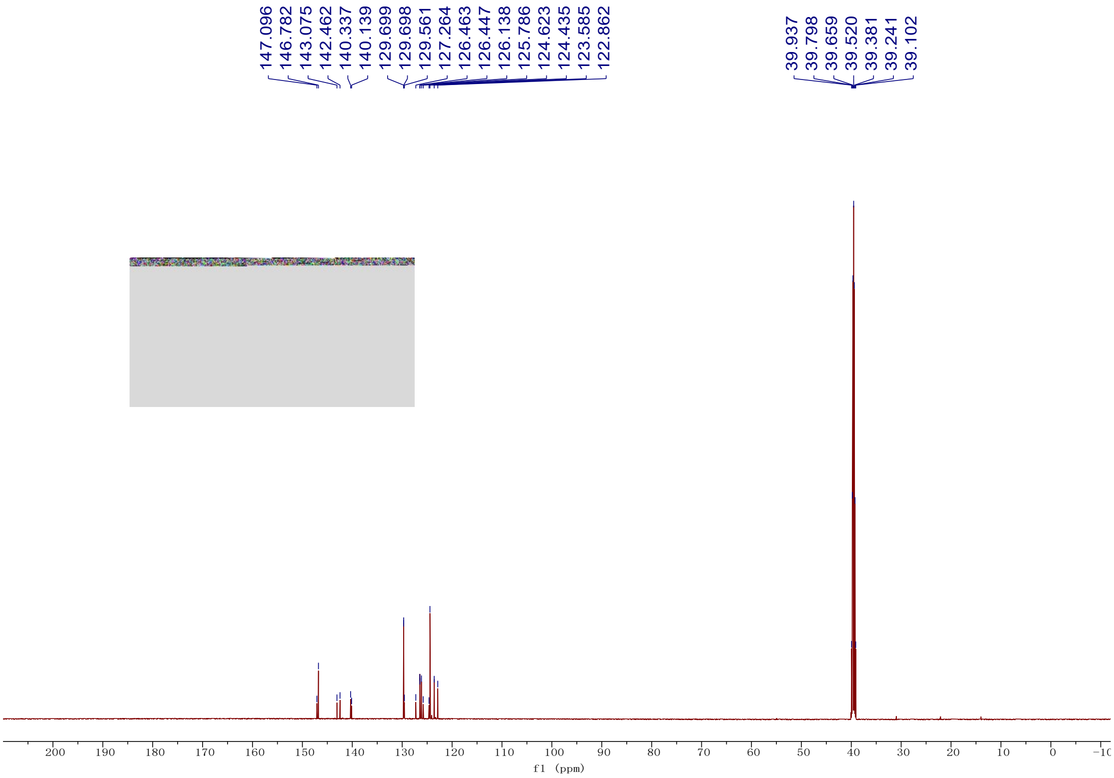

Fig. S5 ^{13}C NMR spectrum of TTVBS.

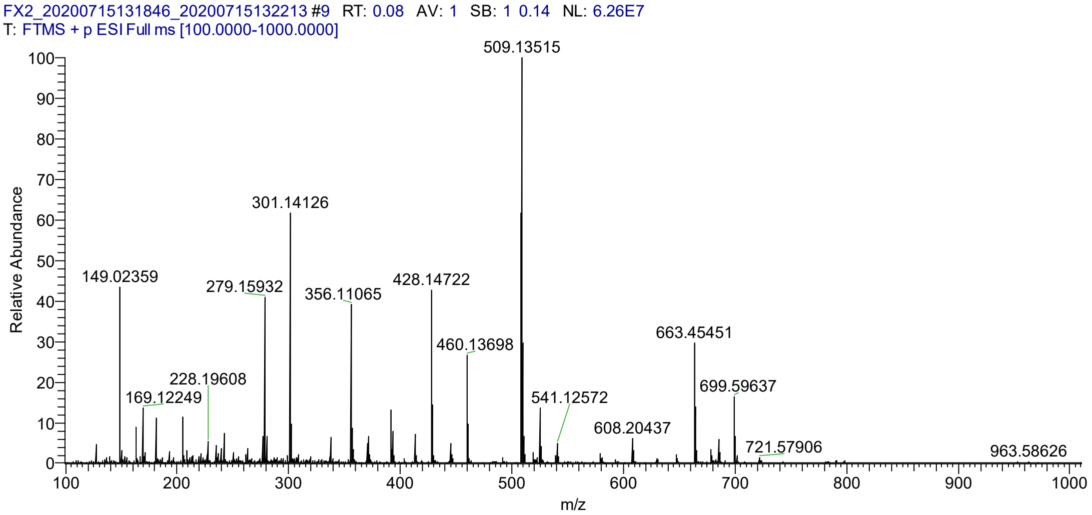

Fig. S6 HRMS spectrum of TTVBS.

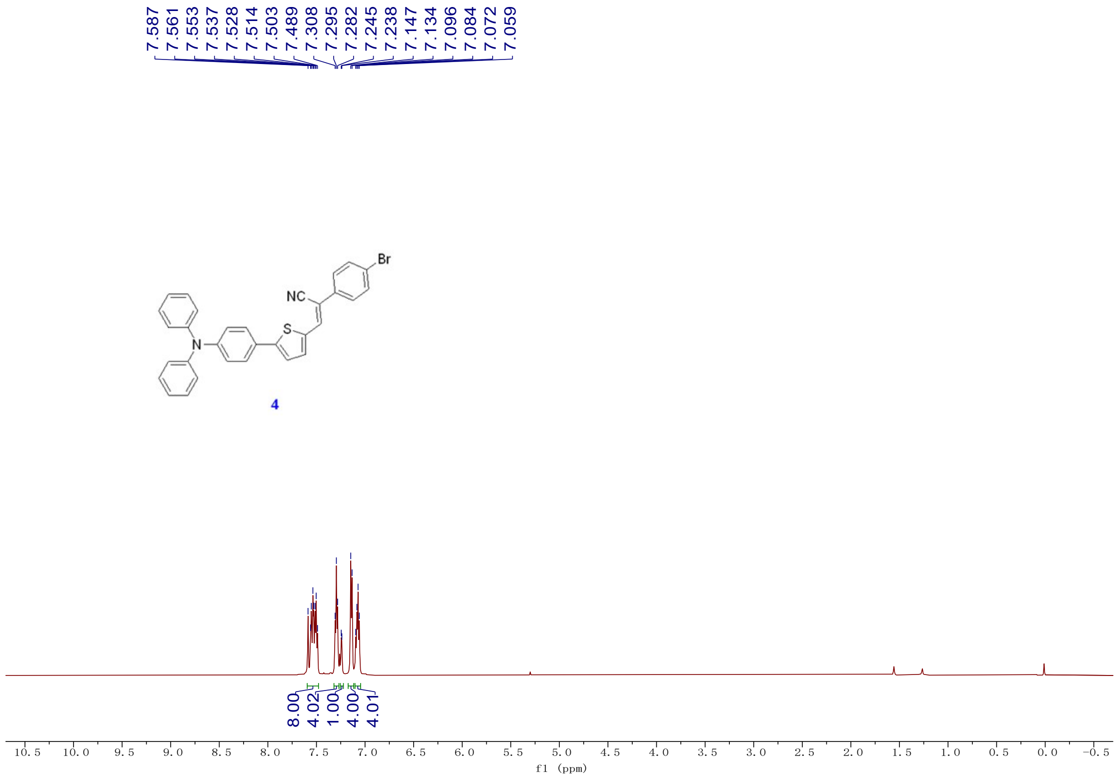

Fig. S7 ^{1}H NMR spectrum of compound **4**.

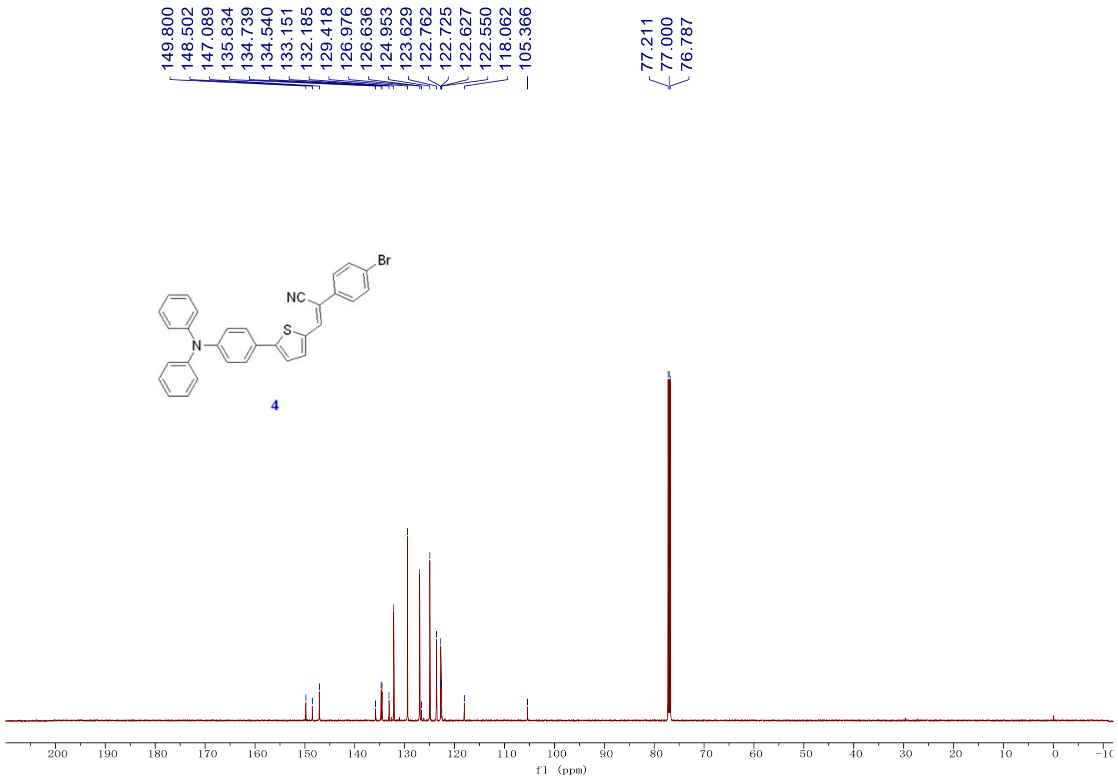

Fig. S8 ^{13}C NMR spectrum of compound **4**.

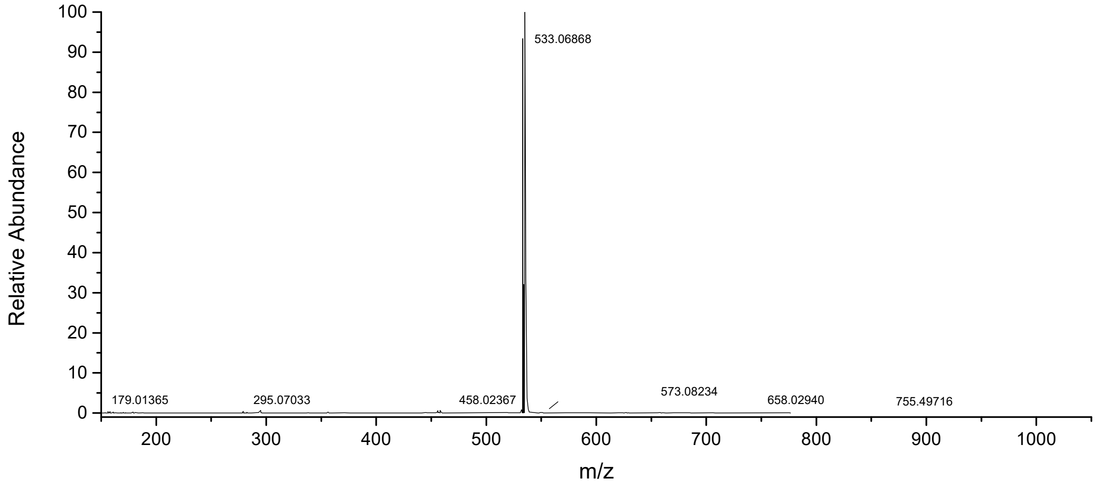

Fig. S9 HRMS spectrum of compound **4**.

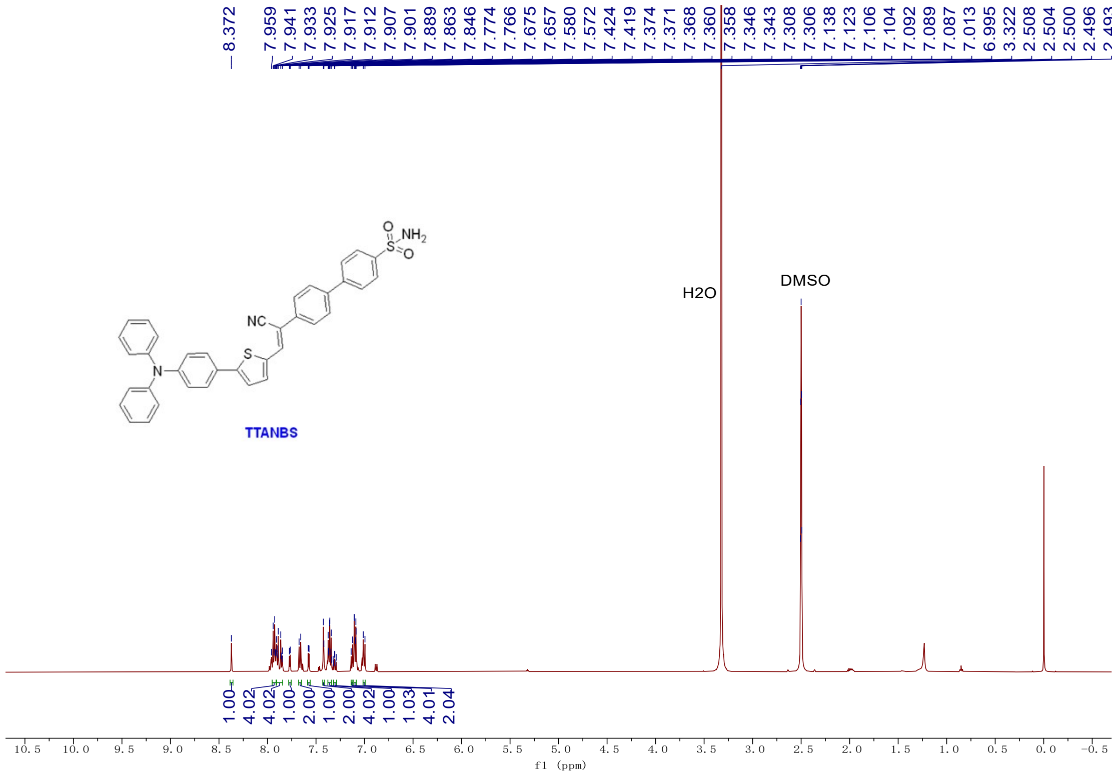

Fig. S10 ^{1}H NMR spectrum of TTANBS.

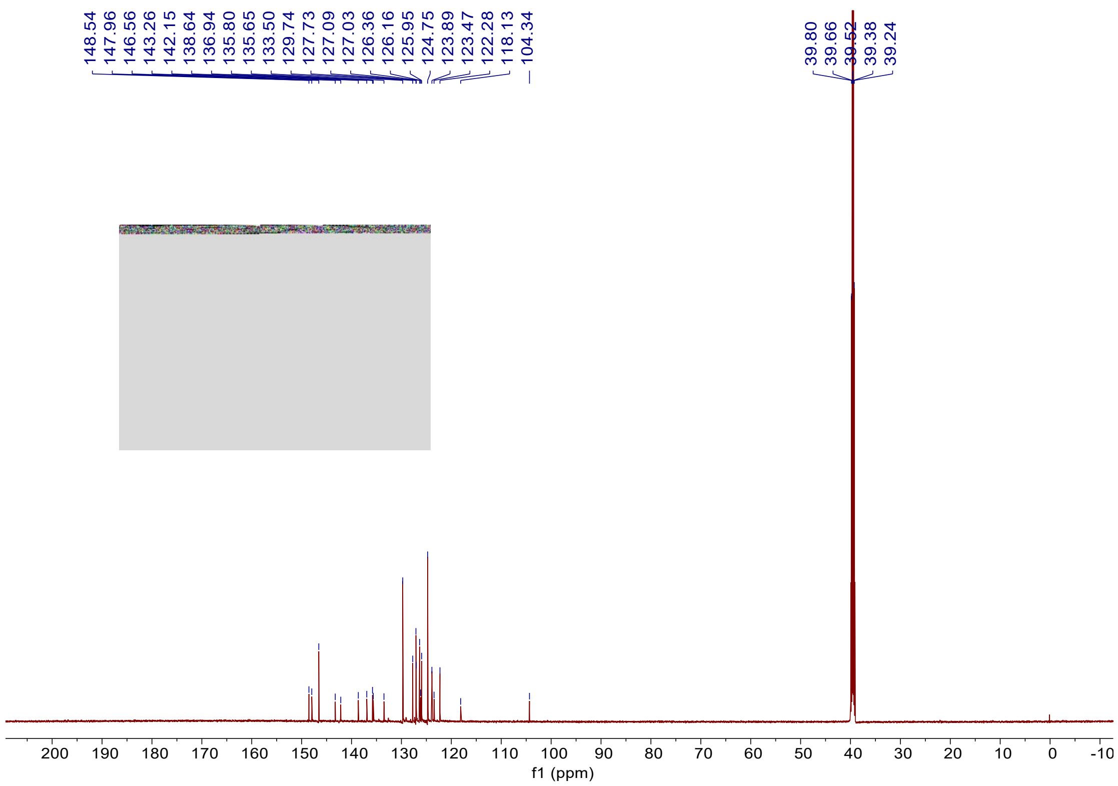

Fig. S11 ^{13}C NMR spectrum of compound **TTANBS**.

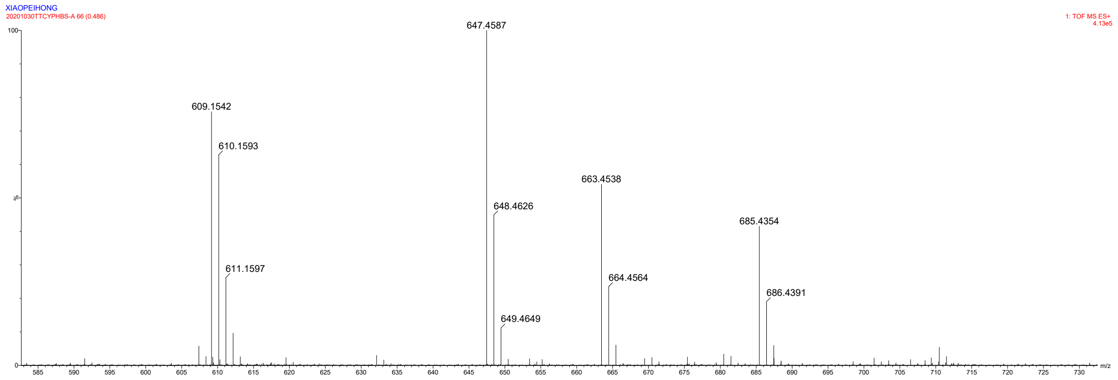

Fig. S12 HRMS spectrum of TTANBS.

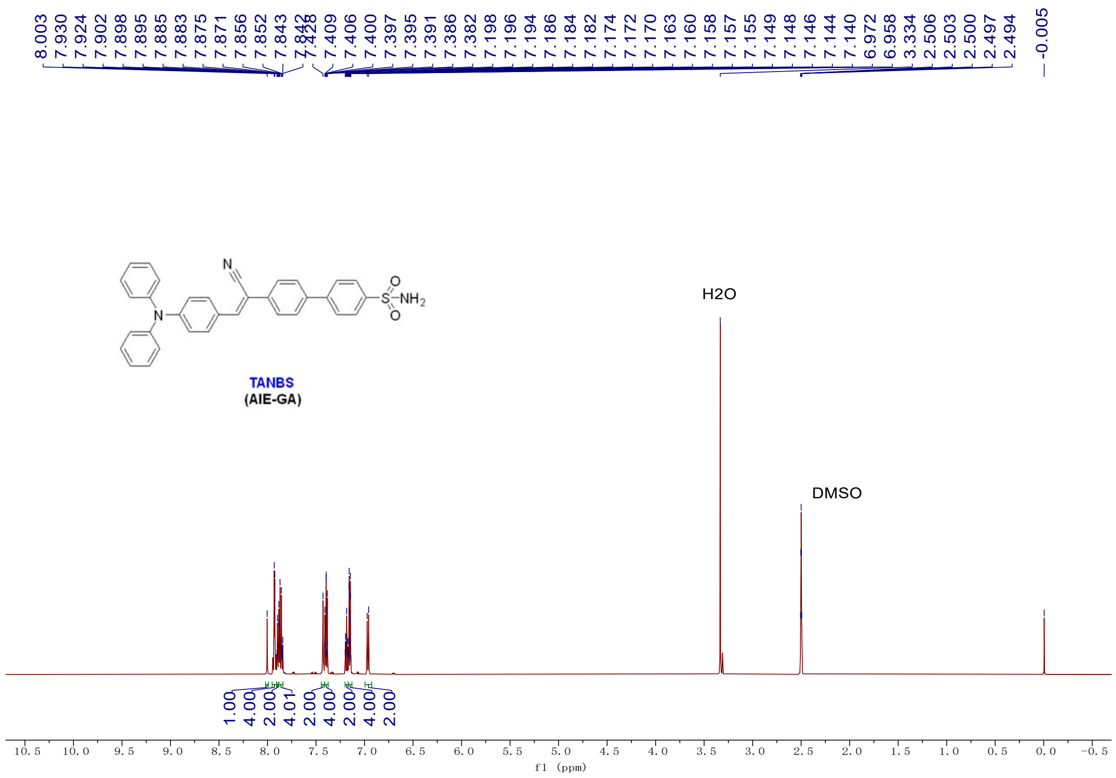

Fig. S13 ^{1}H NMR spectrum of TANBS.

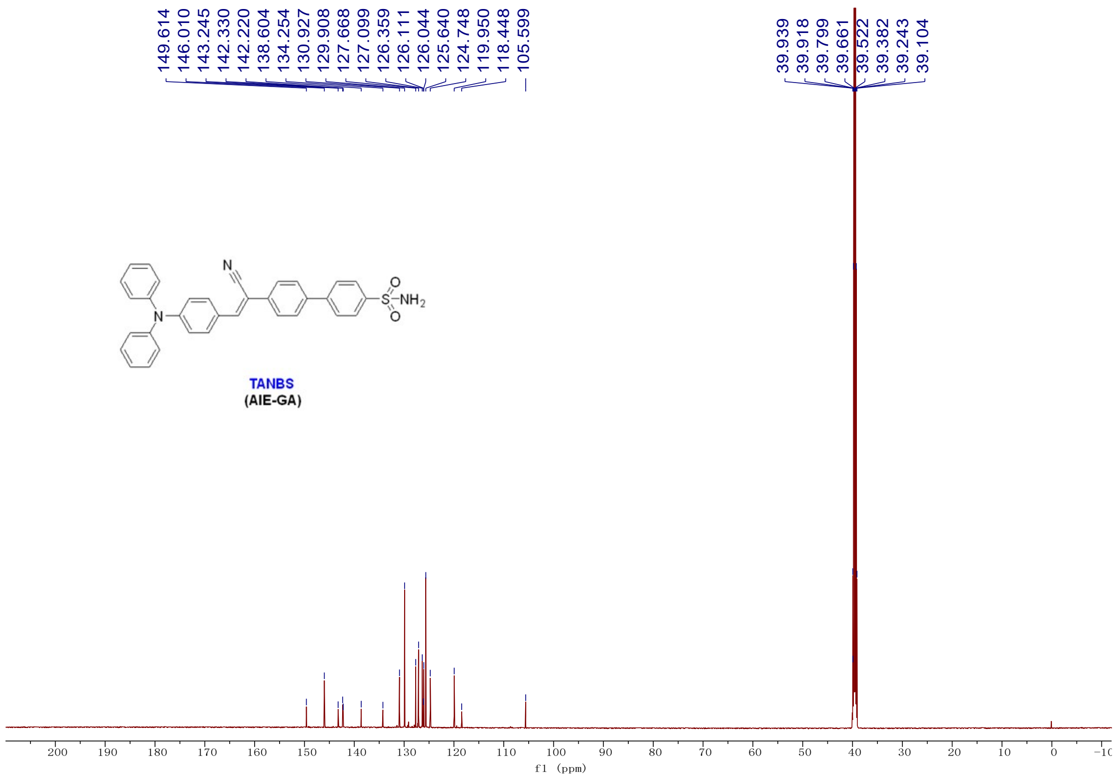

Fig. S14 ^{13}C NMR spectrum of compound **TANBS**.

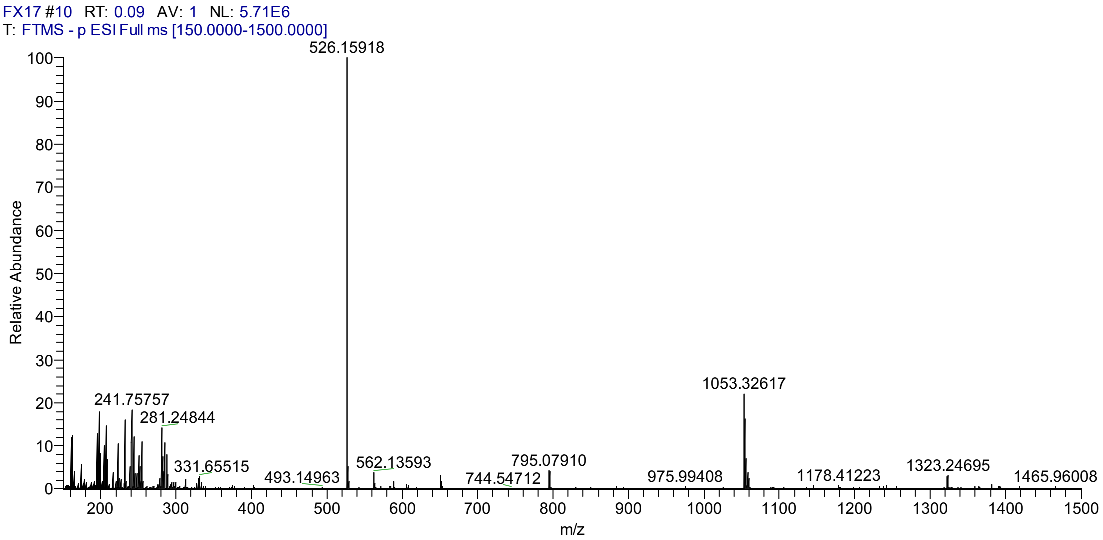

Fig. S15 HRMS spectrum of TANBS.

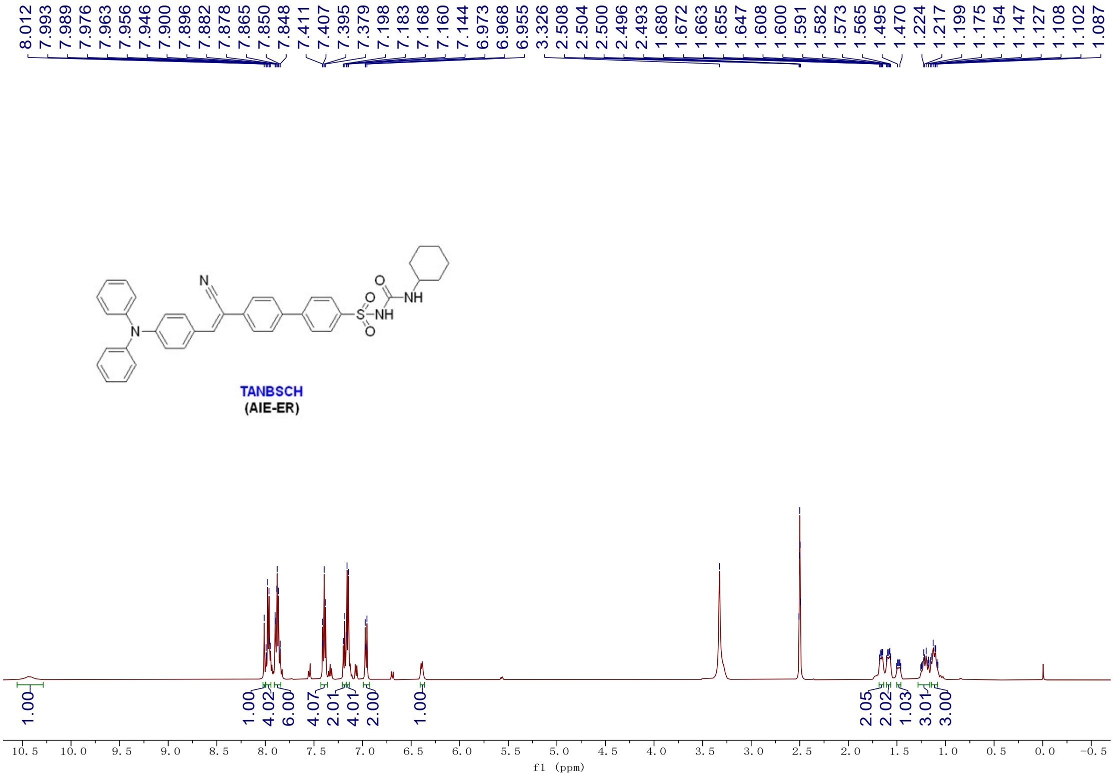

Fig. S16 ^{1}H NMR spectrum of TANBSCH.

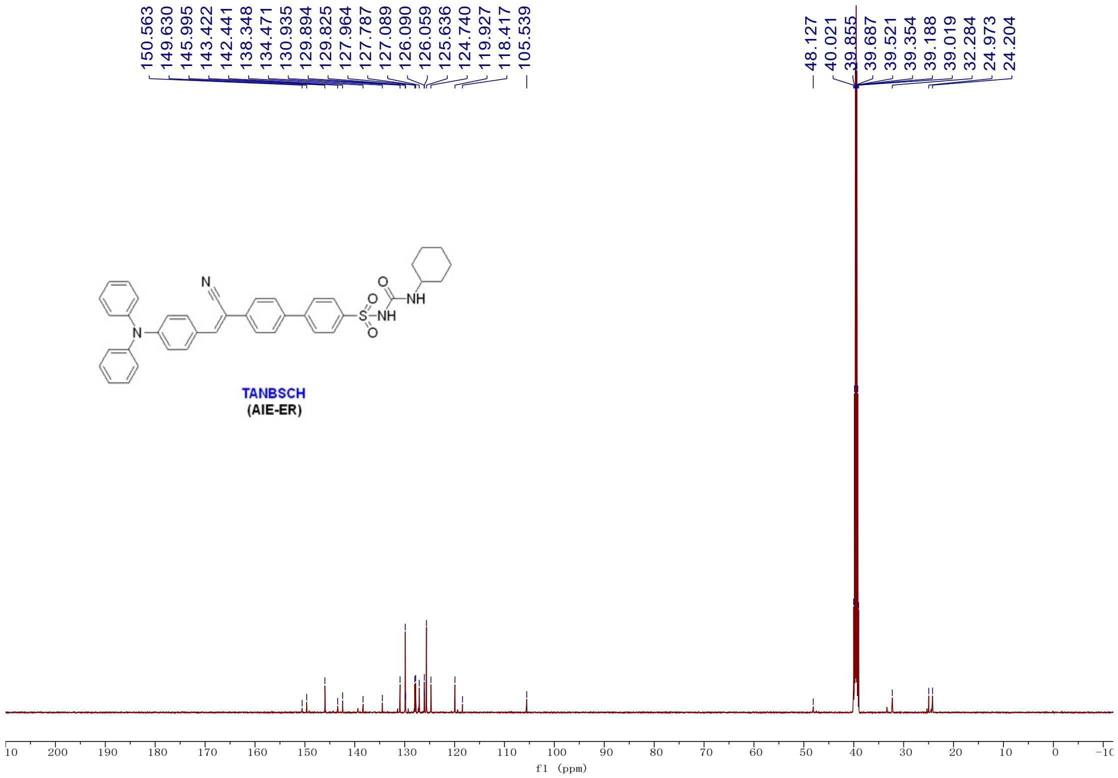

Fig. S17 ^{13}C NMR spectrum of compound **TANBSCH**.

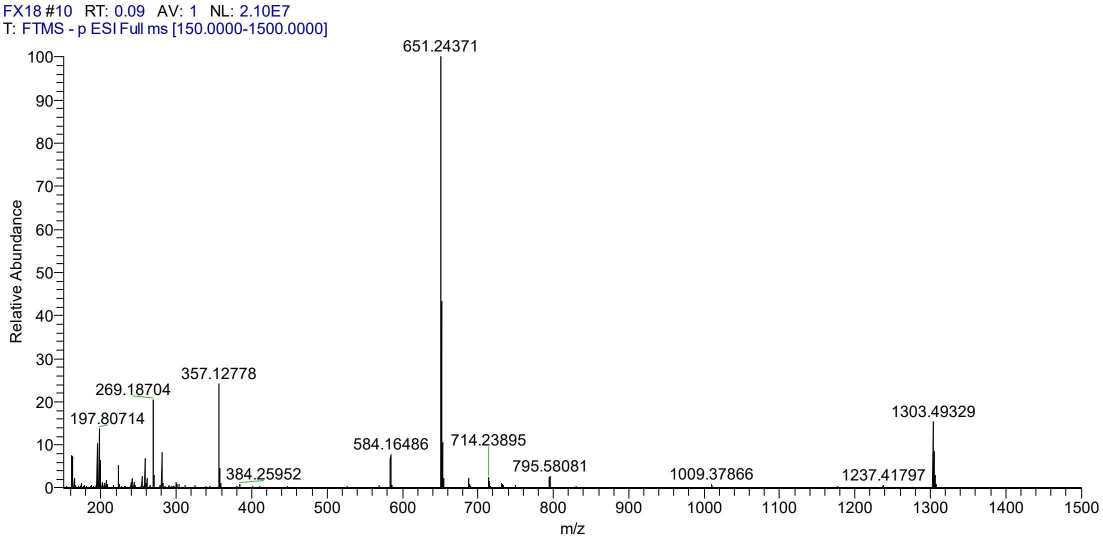

Fig. S18 HRMS spectrum of TANBSCH.

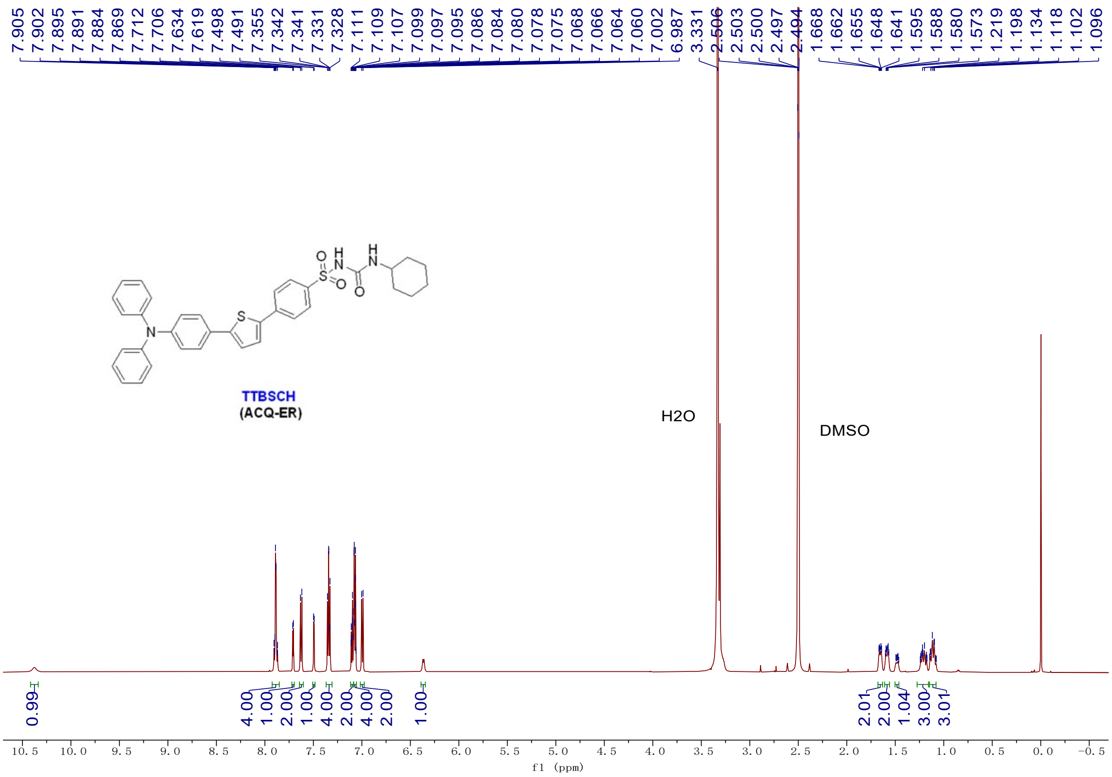

Fig. S19 ^{1}H NMR spectrum of TTBSCH.

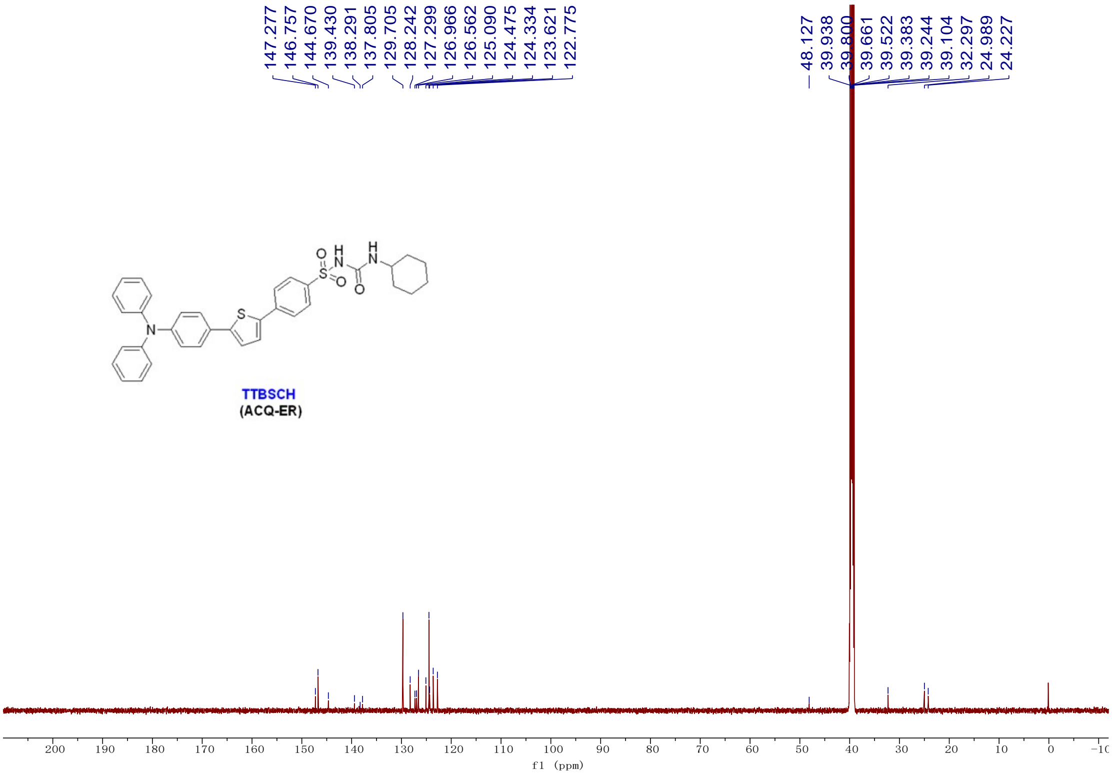

Fig. S20 ^{13}C NMR spectrum of compound **TTBSCH**.

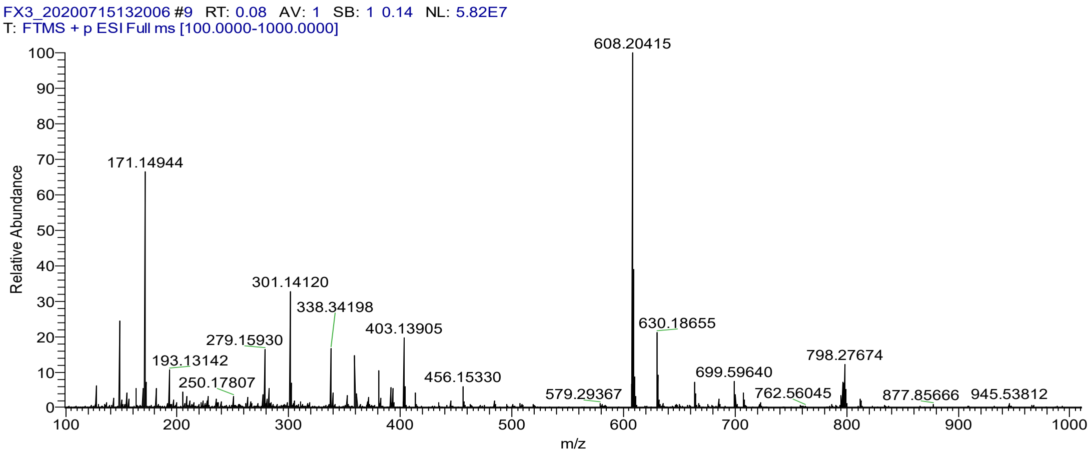

Fig. S21 HRMS spectrum of TTBSCH.

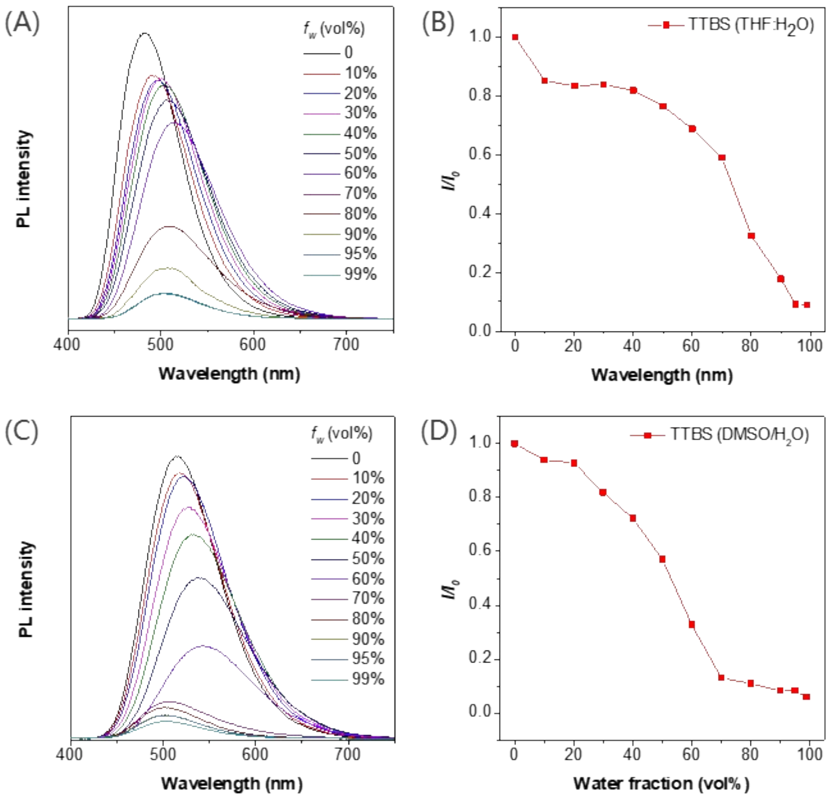

Fig. S22 A) PL spectra of TTBS (10 × 10^{-6} M) in THF/water mixtures with different water fractions (f_w). B) The plot of the relative emission intensity (I/I_0) versus the composition of the THF/water mixture of TTBS. C) PL spectra of TTBS (10 × 10^{-6} M) in DMSO/water mixtures with different water fractions (f_w). D) The plot of the relative emission intensity (I/I_0) versus the composition of the DMSO/water mixture of TTBS.

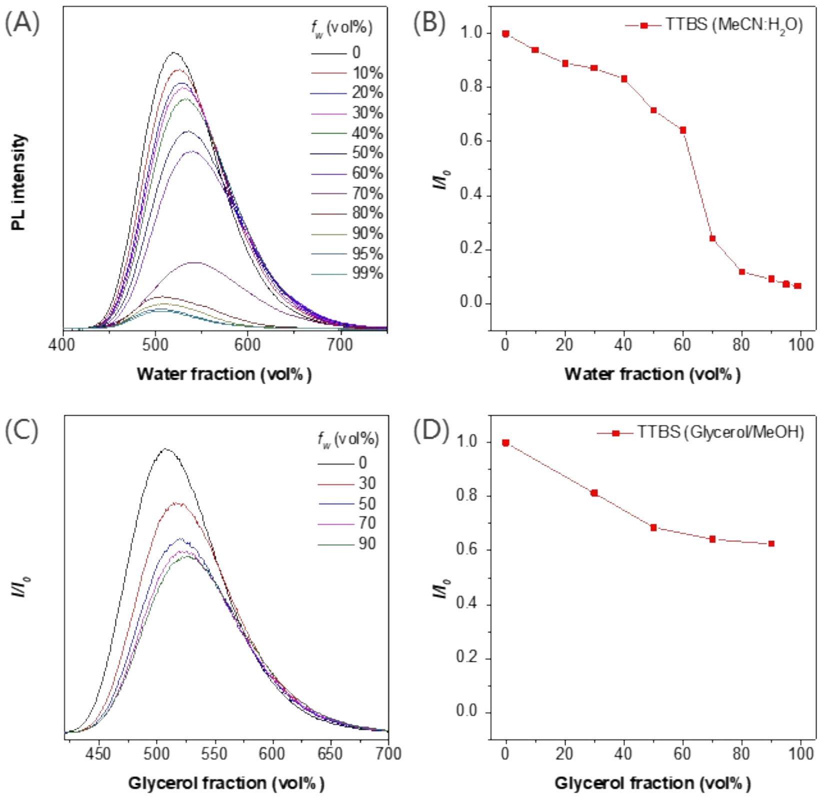

Fig. S23 A) PL spectra of TTBS (10×10^{-6} M) in MeCN/water mixtures with different water fractions (f_w). B) The plot of the relative emission intensity (I/I_0) versus the composition of the MeCN/water mixture of TTBS. C) PL spectra of TTBS (10×10^{-6} M) in MeOH/Glycerol mixtures with different glycerol fractions (f_w). D) The plot of the relative emission intensity (I/I_0) versus the composition of the MeOH/Glycerol mixture of TTBS.

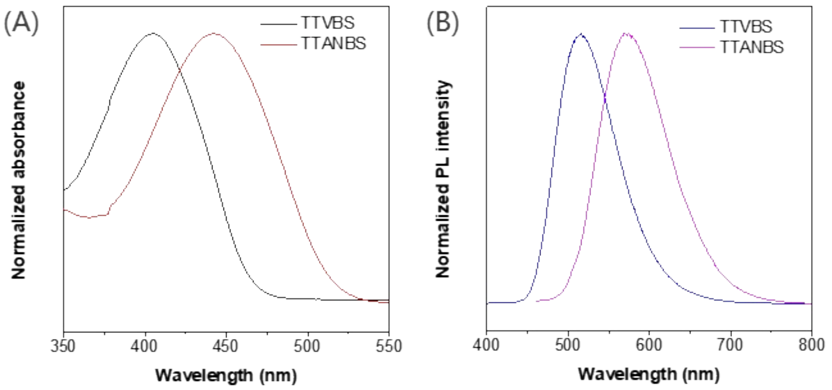

Fig. S24 (A) Normalized absorption and (B) emission spectra of TTVBS and TTANBS in DMSO; concentration = 10×10^{-6} M.

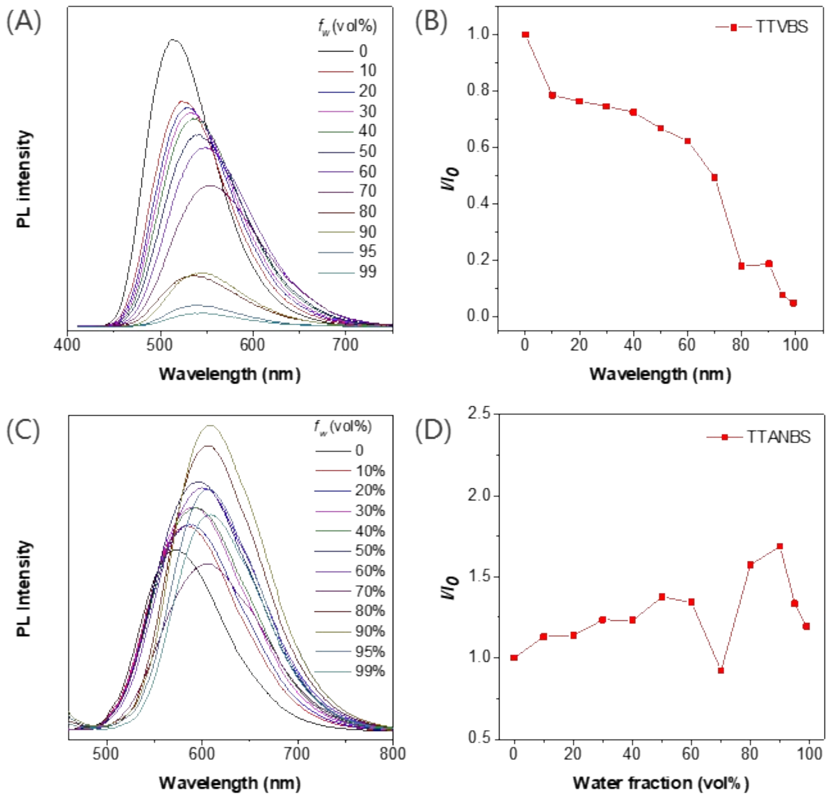

Fig. S25 (A) PL spectra of TTVBS (10×10^{-6} M) in THF/water mixtures with different water fractions (f_w). B) The plot of the relative emission intensity (I/I_0) versus the composition of the THF/water mixture of TTVBS. C) PL spectra of TTANBS (10×10^{-6} M) in THF/water mixtures with different water fractions (f_w). (B) The plot of the relative emission intensity (I/I_0) versus the composition of the THF/water mixture of TTANBS.

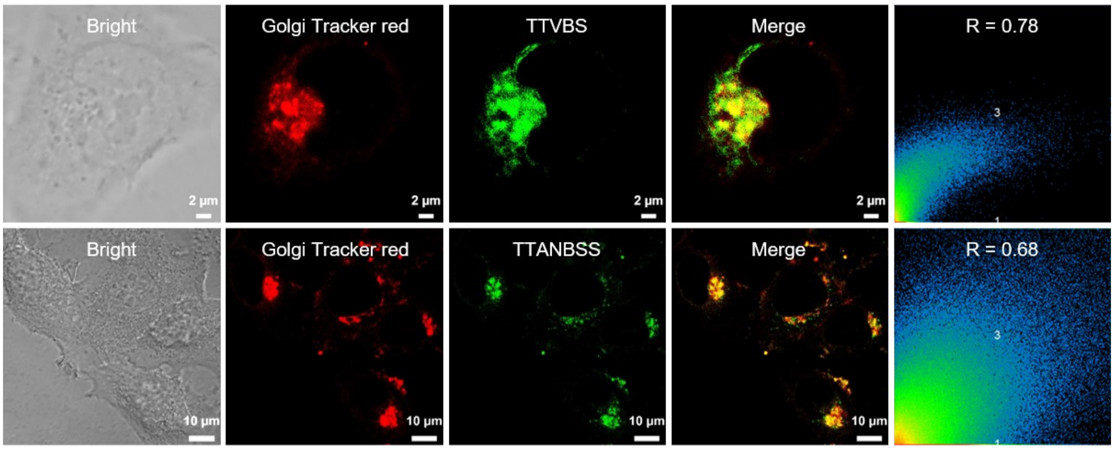

Fig. S26 Confocal microscopy imaging of Hela cells labeled with TTVBS (2×10^{-6} M) and its colocalization with Golgi Tracker Red (333 ug/ml) (R = 0.78); Scale bar = 2 μM (top). TTANBS (2×10^{-6} M) and its colocalization with Golgi Tracker Red (333 ug/ml) (R = 0.68); Scale bar = 10 μM (bottom). TTVBS (green channel: λ_{ex} = 405 nm, λ_{em} = 415 - 600 nm); TTANBS (green channel: λ_{ex} = 405 nm, λ_{em} = 500 - 700 nm); Golgi Tracker Red (red channel: λ_{ex} = 589 nm, λ_{em} = 600 - 700 nm).

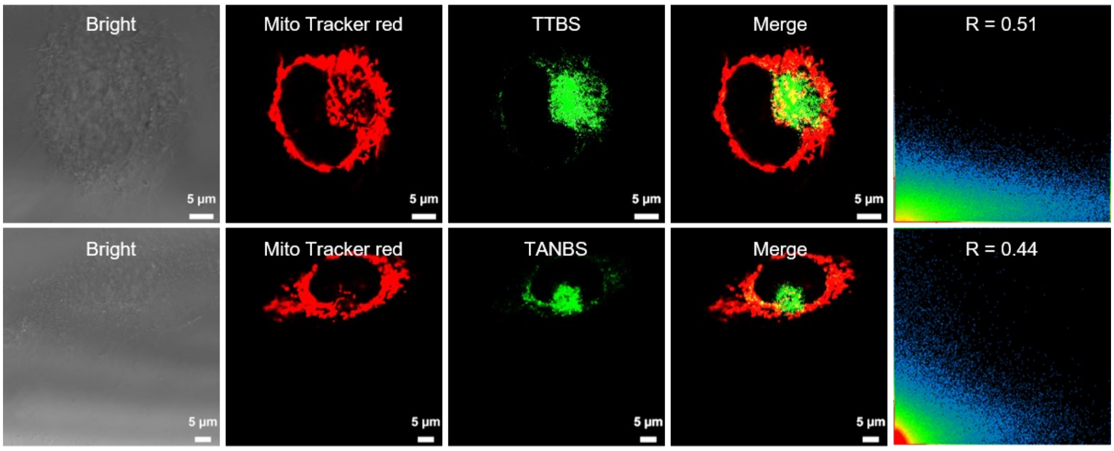

Fig. S27 Confocal microscopy imaging of Hela cells labeled with TTBS (2 x 10^{-6} M) and its colocalization with Mito Tracker Red (100 x 10^{-9} M) (R = 0.51); Scale bar = 5 μM (top). TANBS (2 x 10^{-6} M) and its colocalization with Mito Tracker Red (100 x 10^{-9} M) (R = 0.44); Scale bar = 5 μM (bottom). TTBS (green channel: λ_{ex} = 405 nm, λ_{em} = 415 - 600 nm); TANBS (green channel: λ_{ex} = 405 nm, λ_{em} = 500 - 700 nm); Mito Tracker Red (red channel: λ_{ex} = 579 nm, λ_{em} = 590 - 700 nm).

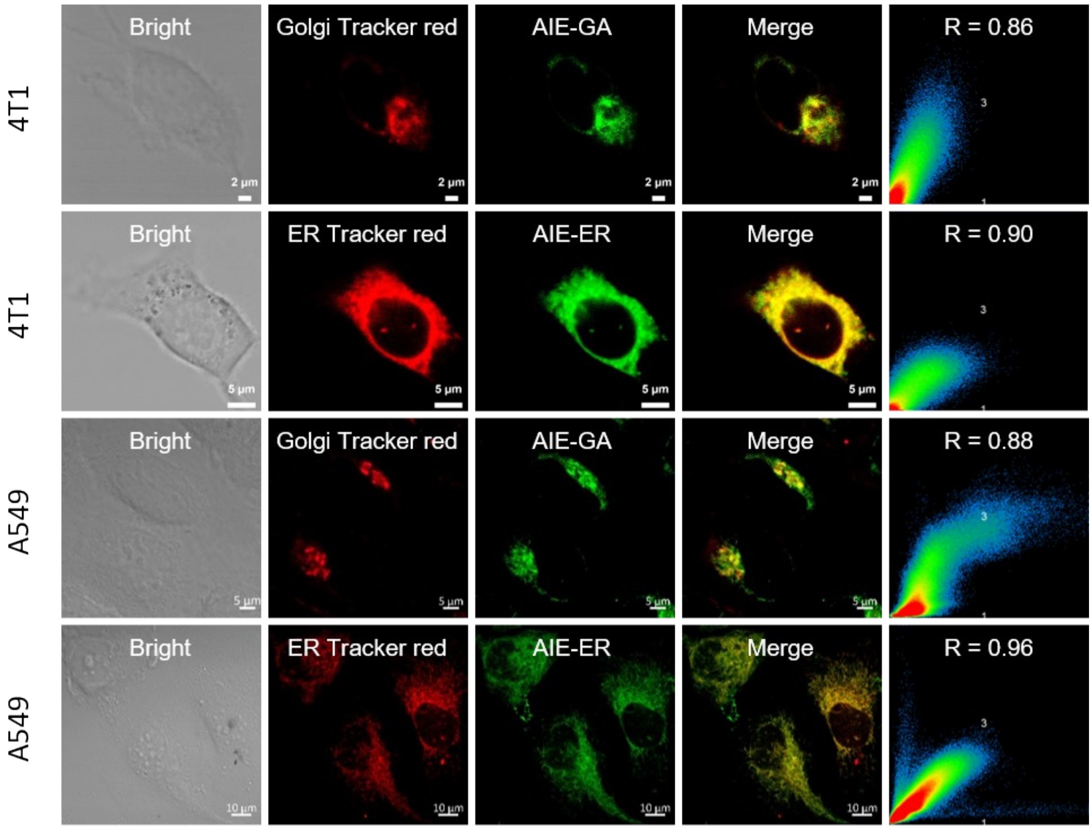

Fig. S28 Confocal microscopy imaging of 4T1 cells labeled with AIE-GA (2 x 10^{-6} M) and its colocalization with Golgi-Tracker Red (333 ug/ml); Pearson's coefficient (R) = 0.86; Scale bar = 2 μM. AIE-ER (1 x 10^{-6} M) and its colocalization with ER-Tracker Red (1 x 10^{-6} M); Pearson's coefficient (R) = 0.90; Scale bar = 5 μM. Confocal microscopy imaging of A549 cells labeled with AIE-GA (2 x 10^{-6} M) and its colocalization with Golgi-Tracker Red (333 ug/ml); Pearson's coefficient (R) = 0.88;

Scale bar = 5 μM. AIE-ER (1 x 10^{-6} M) and its colocalization with ER-Tracker Red (1 x 10^{-6} M); Pearson's coefficient (R) = 0.96; Scale bar = 10 μM. AIE-GA (green channel: λ_{ex} = 405 nm, λ_{em} = 500 - 700 nm); Golgi Tracker Red (red channel: λ_{ex} = 589 nm, λ_{em} = 600 - 700 nm); AIE-ER (green channel: λ_{ex} = 405 nm, λ_{em} = 450 - 650 nm); ER Tracker Red (red channel: λ_{ex} = 543 nm, λ_{em} = 570 - 650 nm).

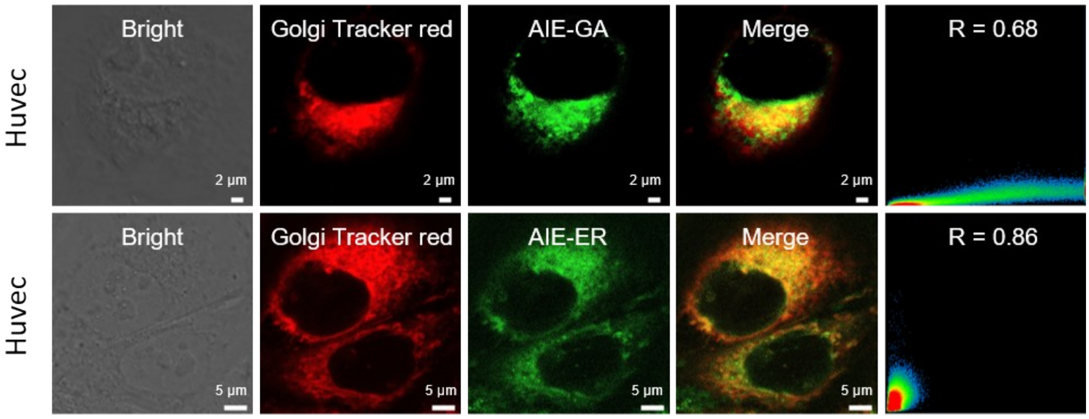

Fig. S29 Confocal microscopy imaging of Huvec cells labeled with AIE-GA (2 x 10^{-6} M) and its colocalization with Golgi-Tracker Red (333 ug/ml); Pearson's coefficient (R) = 0.68; AIE-GA (green channel: λ_{ex} = 405 nm, λ_{em} = 500 - 700 nm); Golgi Tracker Red (red channel: λ_{ex} = 589 nm, λ_{em} = 600 - 700 nm); Scale bar = 2 μM. AIE-ER (1 x 10^{-6} M) and its colocalization with ER-Tracker Red (1 x 10^{-6} M); Pearson's coefficient (R) = 0.86; AIE-ER (green channel: λ_{ex} = 405 nm, λ_{em} = 450 - 650 nm); ER Tracker Red (red channel: λ_{ex} = 543 nm, λ_{em} = 570 - 650 nm); Scale bar = 5 μM.

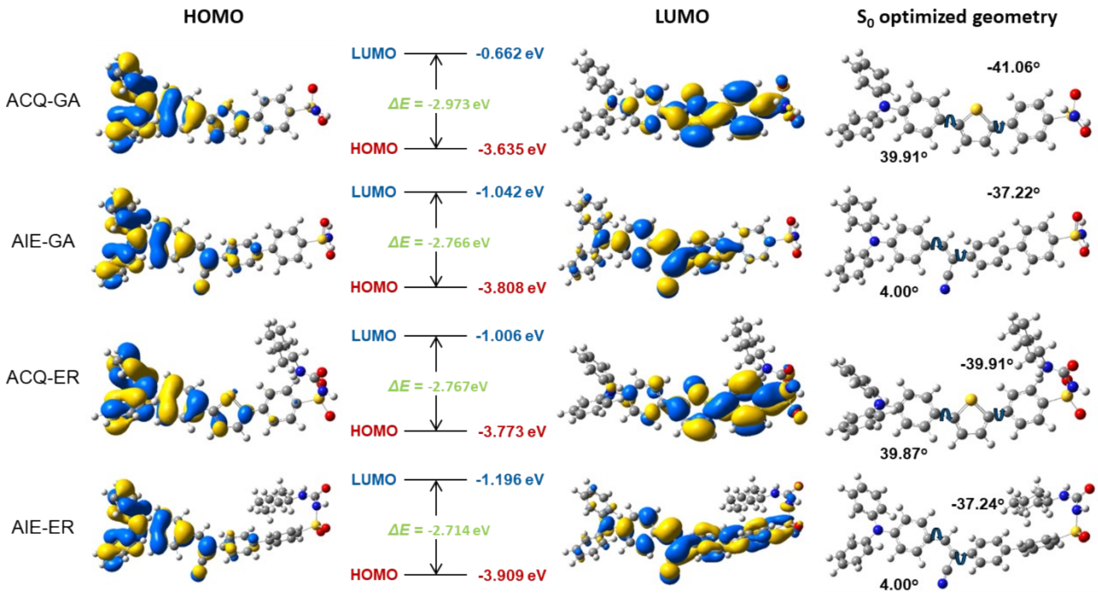

Fig. S30 Molecular orbital amplitude plots of the HOMO and LUMO energy levels of ACQ-GA, AIE-GA, ACQ-ER and AIE-ER.

Table S1. Optical properties of AIE-GA, AIE-ER, ACQ-GA and ACQ-ER.

Probes	λ_{abs} [a] (nm)	λ_{em} (nm) Soln (Φ_F) [b, c]	Aggr	Solid (Φ_F) [c, d]	α_{AIE} ($I_{aggr, max}/I_{soln}$)
AIE-GA	420	521 (0.1%)	548	560 (5.5%)	2.3
AIE-ER	403	527 (0.1%)	545	538 (9.2%)	4.6
ACQ-GA	393	483 (92.9%)	/	530 (11.6%)	/
ACQ-ER	384	500 (93.9%)	/	504 (17.9%)	/

[a] Absorption maximum in DMSO solutions. [b] Emission maximum in THF (10 μM). [c] Fluorescence quantum yield determined by a calibrated integrating sphere. [d] Emission maximum in solid state.
